# A face recognition software framework based on principal component analysis

**DOI:** 10.1371/journal.pone.0254965

**Published:** 2021-07-22

**Authors:** Peng Peng, Ivens Portugal, Paulo Alencar, Donald Cowan

**Affiliations:** University of Waterloo, Waterloo, ON, Canada; Politechnika Slaska, POLAND

## Abstract

Face recognition, as one of the major biometrics identification methods, has been applied in different fields involving economics, military, e-commerce, and security. Its touchless identification process and non-compulsory rule to users are irreplaceable by other approaches, such as iris recognition or fingerprint recognition. Among all face recognition techniques, principal component analysis (PCA), proposed in the earliest stage, still attracts researchers because of its property of reducing data dimensionality without losing important information. Nevertheless, establishing a PCA-based face recognition system is still time-consuming, since there are different problems that need to be considered in practical applications, such as illumination, facial expression, or shooting angle. Furthermore, it still costs a lot of effort for software developers to integrate toolkit implementations in applications. This paper provides a software framework for PCA-based face recognition aimed at assisting software developers to customize their applications efficiently. The framework describes the complete process of PCA-based face recognition, and in each step, multiple variations are offered for different requirements. Some of the variations in the same step can work collaboratively and some steps can be omitted in specific situations; thus, the total number of variations exceeds 150. The implementation of all approaches presented in the framework is provided.

## Introduction

Face recognition has been the subject of research for many years and has been used in countless applications in many different areas. For example, in 2012, Samsung released a new smart TV with a face recognition feature in its built-in camera. This new feature eliminates the need of a userid and password when logging in to social network applications, such as Facebook, Twitter, or Skype. In addition, government interest in face recognition technologies has increased because of its high security level and accessibility. For instance, the US’s Defense Advanced Research Projects Agency (DARPA) expressed interest in replacing traditional digital passwords with a face recognition approach by scanning human faces [1]. Even law enforcement operations can be assisted with the use of face recognition techniques. Karl Ricanek Jr. worked on the detection of potential child pornography in computers using face recognition methods. Speed and accuracy had significant progress as shown in their results [2]. Face recognition is also being used to aid visually impaired individuals to receive information about identity and facial expressions of friends. The authors conducted several studies to create an accessibility bot that runs on the phone and is able to describe face information to users [3]. Lastly, a large application of face recognition is in the social network area. The combination of face recognition, machine learning, and big data has challenges, opportunities, and promising results that might be seen in the near future [4]. It is therefore important to enable the development of applications involving techniques such as machine learning (Principal Component Analysis (PCA) [5], neural networks [6], etc.).

Face recognition application and research has its origins in research in 1964, by Helen Chan and Charles Bisson [7]. Previous to that, research generally looked into the detection of individual features, such as eyes, nose, and mouth. With the development of mathematical approaches, researchers started shifting their foci on the description of the entire face with statistical methods, leading to further advances in face recognition. Current face recognition methods are usually classified in many types, including: feature-based recognition, appearance-based recognition, template-based recognition, etc. Principal component analysis (PCA), as proposed by Turk et al. in 1991 is still one of the most popular analysis techniques to this day [5]. Several variations of the standard PCA approach have been proposed, each for a specific situation. The PCA property of reducing data dimensionality without losing principal components is the key feature that makes it an object of continuing study.

PCA has been the subject of research for several years and has significantly matured as a consequence. However, some challenges remain: its implementation is time-consuming, particularly when it is required to adapt PCA to different types of data, or to combine it with pre-processing or result generation tasks. OpenCV, a popular image processing toolkit, for example, has libraries for standard PCA algorithms built-in, but these libraries have limited customization options. Furthermore, one needs to consider the multiple variations of PCA, since better results are obtained when the suitable PCA variation is used for extreme situations, such as non-uniform illumination [8], or exaggerated facial expressions [9]. Additionally, associated steps such as face detection and pre-processing also play an important role in terms of the entire face recognition process. Selecting appropriate approaches in each step according to specific situations positively affects the final recognition accuracy.

This paper intends to propose a software framework for PCA-based face recognition aiming at assisting software developers to customize their own applications efficiently. The main research question of this work is: How to support the design and implementation of PCA-based face recognition applications through a framework that captures the variability of the face recognition process and can be customized to enable the development of specific applications? This study has four novel contributions. First, it describes a new model for face recognition using PCA with variations at each step of development, and these variations were not captured in previous models. Second, it presents a unique high-level design framework for face recognition application development that allows a general design to be customized to produce specific applications based on selected design variations. Third, it presents the implementation of the framework, which helps developers when choosing a suitable approach for each step of the PCA-based face recognition development process by fostering component reuse. The implementation provides an easier and faster way to extend the framework by reusing existing code components. And fourth, it presents four case studies with applications of different types that can be developed using this study as a supporting tool.

The framework describes the complete process of PCA-based face recognition, and in each step, multiple variations are offered for different requirements. Through different combinations of these variations, at least 108 variations can be produced by the framework. Moreover, some of the variations in the same step can work collaboratively and some steps can be omitted in specific situations; thus, the total of variations exceeds 150. The implementation of all approaches in the framework is provided.

With the framework, software developers working on face recognition applications are able to build their applications quickly through software reuse, as the task becomes a design process at a higher level. After clarifying the requirements of the applications, the framework helps developers to select appropriate variations for each step in the face recognition system. As the framework describes the entire PCA-based face recognition process and demonstrates what type of situations are dealt by the variations, developers simply choose a variation for each step according to the guide of the framework and then build their application.

As an example, if the developer intends to build a face recognition application used for security which works on a high-performance computer, the framework will prioritize the recognition accuracy, whereas the responding speed becomes to a minor factor, since the high performance computer is able to provide enough computation resources. However, when the face recognition is used for smart phones, providing real-time feedback to users is more important, and some extreme environmental conditions such as non-uniform illumination need to be considered. Thus, the framework provides variations which generate results fast and can deal with different working environments.

The paper presents four case studies based on the variations produced by the framework. The first case study is a face recognition system for smart phones. The other three case studies aim to cover all variations to give a comprehensive impression of the framework to readers. For instance, the Case Study 2 describes a face recognition application working on high performance computers. However, the possible applications which can be produced by the framework are not limited to the case studies.

### Related areas

#### Face recognition system

Currently, personal identification still heavily relies on traditional password encryption. This method do help people protect their privacy; however, with the development of other high-tech fields, the security level provided by a password is not able to meet our requirements, as it is based on “what the person possesses” and “what the person remembers”, instead of “who the person is”. Fortunately, a new research area, biometric recognition, offers a number of technical methods, which may make truly reliable personal identification come true.

In the field of biometrics recognition, face recognition is the friendliest, most direct and natural method. Compared with other recognition approaches, such as fingerprint recognition or iris recognition, face recognition does not invade personal privacy or disturb people. Additionally, a face image is easier to capture, even without making the person aware that an image is being made.

According to a report from Research and Markets, Asia and North America are the two regions with the most recent advances in the face recognition area. In addition, the global face recognition market in 2017 was worth 3.85 billion USD, with future projections that reach 9.78 billion USD in 2023 [10].

Generally, an automatic face recognition system is divided into phases, face detection and face recognition. In the face detection stage, the face area is extracted from the background image, and the size of the area is also defined at the same time. In the face recognition stage, the face image will be represented with mathematical approaches to express as much information about the face as possible. Eventually, the new face image will be compared with known face images, which results in a similarity score for final verification.

Thanks to a human being’s eyes, the aforementioned two phases can be easily completed. However, building an automatic face recognition system with high accuracy is challenging, as every phase in the recognition process is susceptible to internal physiological and external environmental factors. Therefore, face recognition is still attracting researchers.

#### Principal component analysis

The earliest principal component analysis dates back to 1901 when Karl Pearson proposed the concept and applied it to non-random variables [11]. In 1930, Harold Hotelling extended it to random variables [12, 13]. The technique is now being applied in a number of fields, such as mechanics, economics, medicine, and neuroscience. In computer science, PCA is utilized as a data dimensionality reduction tool. Especially in the age of Big Data, the data we process is often complex and huge. So reducing the computational complexity and saving computing resources are important issues.

Basically, the PCA process projects the original data with high dimensionality to a lower dimensionality subspace through a linear transformation. Nevertheless, the projection is not arbitrary. It has to obey a rule that the most representative data needs to be retained, i.e. the data after transforming cannot be distorted. Hence, those dimensionalities which are reduced by PCA are actually redundant or even noisy. Therefore, the ultimate goal of conducting PCA is to refine data so that the noisy and redundant part can be removed and only the useful part is retained. It is because of this feature that PCA is widely used in face recognition. Images are represented as a high dimensional matrix in computers, and removing noise from images is a necessary pre-processing step.

#### Object-oriented framework

An object-oriented framework is a group of correlated classes for a specific domain of software. It defines the architecture of a class of user applications, the separation of object and class, the functionality of each part, how the object and class collaborate, and the controlling process. Therefore, one focus of an object-oriented framework is software reusability [14].

Software reuse uses existing knowledge of a software to build a new software, so that to reduce the cost of development and maintainence. In 1992, Charles W. Krueger suggested five dimensions for a good software reuse, which are abstraction and classification in terms of building for software reuse process, and selection, specialization, and integration in terms of building with software reuse process. Abstraction and classification means that in software reuse, the reusable knowledge should be represented concisely and classified. Selection, specialization and integration indicate that reusable knowledge should be parameterized for query, specialized for new situations, and integrated for customer projects [15].

A framework can be viewed as the combination of abstract class and concrete class. The abstract class is defined in the framework, whereas the concrete class is implemented in the application. Simply, a framework is the outline of an application, which contains the common objects for a specific domain. In addition, a framework includes some design parameters, which can be used as interfaces, to be applied to different applications.

#### Machine learning approaches

Machine learning is an interdisciplinary subject consisting of many different areas, such as probability, statistics, approximation theory, and algorithm theory. Arthur Samuel first defined machine learning as a “field of study that gives computers the ability to learn without being explicitly programmed” [16].

Machine learning focuses on simulating human beings’ behaviors to gain new knowledge and skills with a computer. Furthermore, it is able to recombine the learned knowledge and keep improving its performance.

Typically, machine learning is classified into three categories, which are supervised learning, unsupervised learning, and reinforcement learning [17]. The difference mainly depends on whether the computer is taught or not. In supervised learning, the computer is given input along with its corresponding output. However, in unsupervised learning, no labels are provided, so the computer needs to learn on its own. Unsupervised learning does not always have an explicit goal, which means that it is allowed to find a goal by itself. Reinforcement learning can be treated as a compromise between the two aforementioned approaches. It has an explicit goal, but it needs to interact in a dynamic environment in which no teaching is provided.

### Problem

Although there exists a number of image processing toolkits like OpenCV, which have PCA algorithm as well as associated approaches for face recognition, it is still time-consuming for software developers who intend to integrate face recognition implementations with their own applications. Furthermore, selecting appropriate approaches for each step in the process of face recognition is non-trivial, since it directly impacts the final recognition result. For face recognition systems which run under extreme situations, such as non-uniform illumination, exaggerated facial expression, or facial region occlusion, approach selection becomes even more significant. In fact, building a PCA-based face recognition system should not cost a lot of effort for developers, as the technique has been studied for years and is mature. The time spent on implementing the algorithms and integrating with their applications should not be necessary.

### Proposed approach

This paper provides a software framework for PCA-based face recognition aiming at assisting software developers to customize their own applications efficiently. The framework describes the complete process of PCA-based face recognition including image representation, face detection, feature detection, pre-processing, PCA, and verification, and in each step, multiple variations are offered to fit different requirements. Through various combinations of these variations, at least 108 variations can be generated by the framework. Moreover, some of the variations in the same step can work collaboratively and some steps can be omitted in specific situations; thus, the total number of variations exceeds 150. The implementation of all approaches presented in the framework is provided. As the framework strictly follows the normal process of PCA-based face recognition, it can be easily extended, which means more approaches are able to be attached to any of the steps.

### Evaluations

In the paper we present a framework followed by four case studies. The first case study is for face recognition used on smart phones. The other three case studies cover almost every variation supported by the framework.

### Contributions

The main contributions described in this paper are:

1. A model including the entire facial recognition process using PCA, multiple variations for each phase suitable for different facial conditions;
2. A high-level framework design;
3. An implementation of the framework; and
4. A support tool for facial recognition with PCA

### Paper outline

The Related Work section presents work related to the research which mainly includes four subsections. The first subsection introduces general requirements of face recognition system. The second subsection focuses on principal component analysis which is the core algorithm of this research. The third subsection explains the concept of object-oriented frameworks. The last subsection talks about machine learning. Our main section entitled “Framework for PCA-based Face Recognition” demonstrates the framework. The Case Studies section describes the case studies based on the proposed framework. The last section of this paper concludes the paper and suggests future work.

## Related work

As mentioned previously, this section introduces work in four areas related to our research. First, a classical face recognition framework is demonstrated. Then, we present a brief introduction to principal component analysis (PCA) describing the history of the approach, the mathematical principal behind PCA, and its development in face recognition. Third, object-oriented frameworks are discussed. Last, we investigate machine learning approaches, since its outstanding classifying ability has been attracting researchers in face recognition.

### Face recognition framework

**Fig 1:**
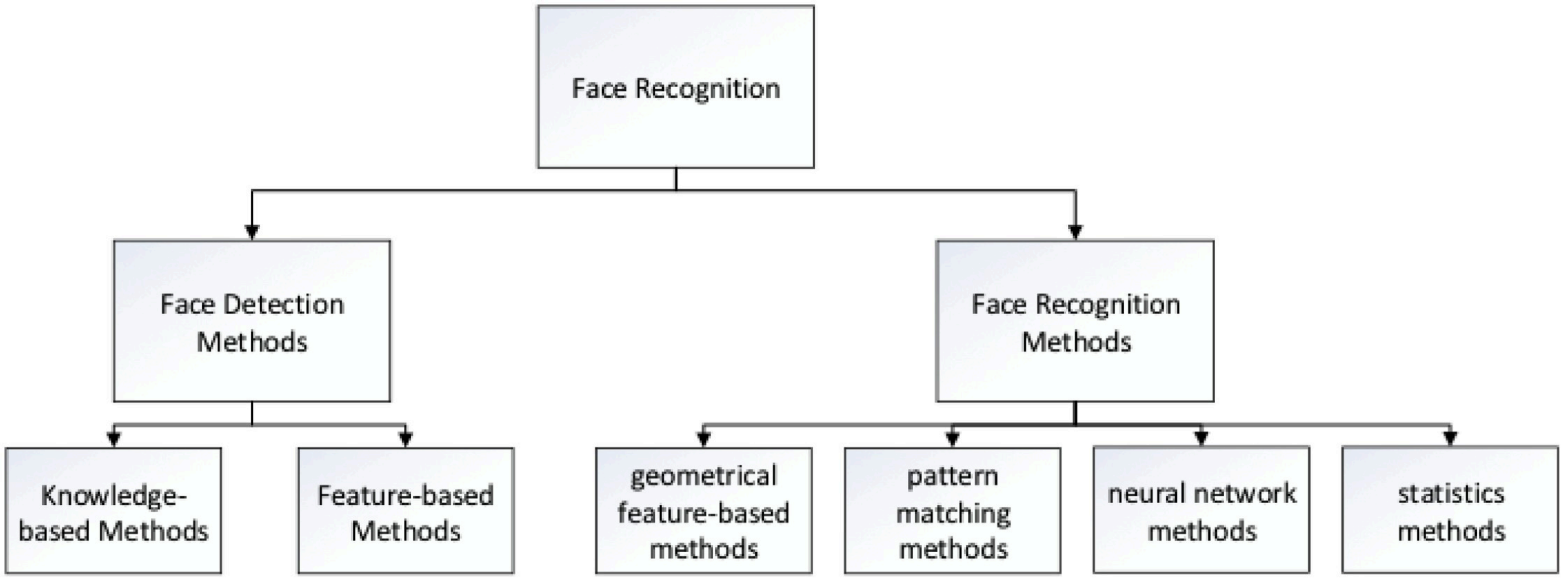
Face recognition system.

Generally, a face recognition framework is divided into two sequential processes, which are face detection and face recognition. As introduced in the previous section, face detection focuses on capturing the face region from the image. Then the face region is delivered to a face recognition process for verification. The structure of this process is shown in Fig 1.

#### Face detection

Face detection is a necessary step in face recognition systems, which localize and extract the face region from the background [18, 19]. Basically, face detection can be classified into two categories, which are knowledge-based methods, and feature-based methods [20].

Knowledge-based methods are actually based on a series of rules generated from researchers’ prior knowledge of human faces, such as the face color distribution, distance or angular relationship between eyes, nose, and mouth. Most of these rules are straightforward and easy to find.

Rai et al. [21] proposed a face detection system for real-time operation in mobile devices. The system is based on OpenCV, a library for real-time computer vision applications, and has layers for image preprocessing and face detection. During preprocessing, Gaussian smoothing reduces image noise and grayscale transformations are applied for improved processing. The preprocessing layer also includes contrast enhancement on grayscale values of image points that have been smoothed, and binarization for feature identification. In the face detection layer, the system searches for Haar-like features, commonly used in face detection applications and native in OpenCV.

Feature-based methods detect face region based on internal facial features as well as the geometrical relationship among them [22]. Contrary to knowledge-based methods, feature-based methods seek constant features as a means of detection. Researchers have proposed a number of methods, which detect face features first, then deduce whether this a real face. Facial features, such as eyebrow, eyes, nose, mouth, and hairline are usually extracted with an edge detector. According to the extracted features, statistical models describing the relationship between each feature can be built, so that the face region can be captured. However, feature-based methods are always susceptible to illumination, noise, and occlusion, as these factors seriously damage edges on face [23].

#### Face recognition

Face recognition methods can be classified into three categories, which are early geometrical feature-based methods and pattern matching methods, neural network methods, and statistical methods [24, 25].

The earliest face recognition was based on geometrical features of a face. Simply, the basic idea of this kind of method is to capture the relative position and relative size of representative facial components, such as eyebrows, eyes, nose, and mouth [26]. Then face contour information is included to classify and recognize the faces. Pattern matching methods are the simplest classification methods in the field of pattern recognition. In face recognition, face images in a dataset are treated as the pattern, so once a new image is available, a correlation score between the pattern and the new image can be calculated to generate the final result.

Artificial neural network research dates to the 1940s when Warren McCulloch and Walter Pitts [27] first applied the concept to mathematics and algorithms. The idea of artificial neural networks is inspired by biological neural networks, which consist of a large number of neurons. The neurons in artificial neural networks are actually a group of individual functions, each of which is responsible for a certain task. The neurons are connected with weighted lines which pre-process the input generated from the previous neuron. The advantages of applying neural network to face recognition are its ability to store distributed data that can be processed in parallel.

The structure of a single neuron is simple with limited functionality; however, an entire neural network consisting of a number of neurons is able to achieve various complicated goals. Furthermore, the most significant feature that neural network possesses is self-adaptability, which means it is able to enhance itself through iteration. The most representative neural network methods in face recognition are multi-level BP networks and RBF networks [28, 29].

**Fig 2:**
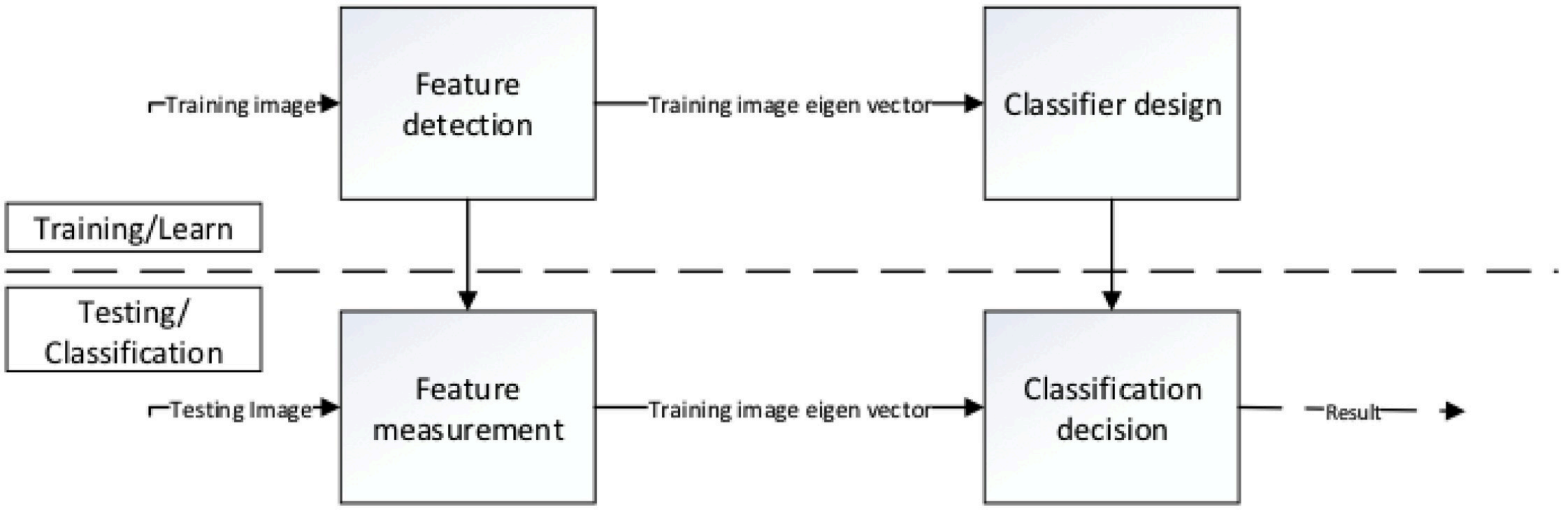
Learning and classification process.

Statistics-based methods attract attention from researchers in face recognition. The idea of a statistics-based method is to capture statistical feature of a face through learning, and then use the acquired knowledge to classify the face. The learn and classification process is shown in Fig 2.

Among all statistics-based methods, subspace analysis is the major type. The basic idea is to compress the face image from a high dimensional space to on with lower dimensions through a linear or non-linear transformation. These methods include Linear Discriminant Analysis (LDA), Independent Component Analysis (ICA), and Principal Component Analysis (PCA).

### Principal component analysis

In computer science, particularly in the context of Big Data, data is often expressed as vectors and matrices. In terms of images, the increase of the resolution of an image means the size of the matrix is larger. Although current computers are powerful enough to process huge amount of data in relatively short time, efficiency still needs to be considered.

Principal component analysis has been widely recognized as an efficient data dimensionality reduction method using a linear transformation [30]. While reducing the data dimensionality, retaining significant information is the basic requirement.

In statistics, mean value, standard deviation, and variance are always used to analyze the distribution and variation of a set of data. These three values can be calculated with Eqs (1), (2) and (3).

$$\begin{array}{c} \bar{x}=\frac{\sum_{i=1}^{n}x_{i}}{n} \end{array}$$ (1)

$$\begin{array}{c} s=\sqrt{\frac{\sum_{i=1}^{n}{\left(x_{i}-\bar{x}\right)}^{2}}{n-1}} \end{array}$$ (2)

$$\begin{array}{c} s^{2}=\frac{\sum_{i=1}^{n}{\left(x_{i}-\bar{x}\right)}^{2}}{n-1} \end{array}$$ (3)

However, mean value, standard deviation, and variance functions only work for one-dimensional data. In computer science, the data is always multi-dimensional. So, a new measurement which conveys a relationship among data of different dimension needs to be included, which is covariance. Normally, covariance is able to describe the relationship between two random variables, as shown in Eq (4).

$$\begin{array}{c} cov\left(X,Y\right)=\frac{\sum_{i=1}^{n}X_{i}-\bar{X}\left(Y_{i}-\bar{Y}\right)}{n-1} \end{array}$$ (4)

Therefore, as the dimension increases, multiple covariances need to be calculated, e.g. the number of covariance needed when dealing with n-dimensional data is shown in Eq (5).

$$\begin{array}{c} \frac{n!}{(n-2)!\times 2} \end{array}$$ (5)

Fortunately, a matrix approach offers a perfect solution for this calculation. The Eq (6) shows the definition of a covariance matrix.

$$\begin{array}{c} C_{n\times n}=\left(c_{i,j},c_{i,j}=cov\left(Dim_{i},Dim_{j}\right)\right) \end{array}$$ (6)

Eq (7) shows the covariance matrix of a dataset with three dimensions {*x*, *y*, *z*}.

$$\begin{array}{c} C=(\begin{array}{ccc} cov(x,x) & cov(x,y) & cov(x,z)\\ cov(y,x) & cov(y,y) & cov(y,z)\\ cov(z,x) & cov(z,y) & cov(z,z) \end{array}) \end{array}$$ (7)

It can be found that covariance matrix is a symmetric matrix, whose diagonal shows the variance of each dimensions.

After generating the covariance matrix, we are able to calculate its eigenvalues and eigenvectors through Eq (8).

$$\begin{array}{c} A\alpha =\lambda \alpha \end{array}$$ (8)

Where *A* stands for the original matrix, λ stands for an eigenvalue of *A*, and *α* represents the eigenvector according to eigenvalue λ. Usually eigenvalues are sorted in descending order, which corresponds to the importance of the eigenvector. We can choose how much information to retain. In this case, selecting a good threshold with which useful information is retained, whereas less significant information is removed, becomes important.

### Object-oriented frameworks

In recent years, software reuse has become a significant technique in software engineering. Traditional methods, such as function or library, provide limited reuse, whereas object-oriented frameworks aim at larger components, such as business units and application domains. Building object-oriented framework can save users countless hours and thousands of dollars in development costs by providing reusable skeletons [31]. Object-oriented framework development plays an increasingly necessary role in contemporary software development [32, 33]. Frameworks like MacApp, ET++, Interviews, ACE, Microsoft’s MFC and DCOM, JavaSoft’s RMI, and implementations of OMG’s CORBA are widely used [34] Some of the features of object-oriented framework are listed below:

A. Modularity
  Framework enhances software modularity by encapsulating variable implementation details into fixed interfaces. The impact caused by variations of design and implementation is localized by a framework, so that makes software maintenance much easier.
B. Reusability
  Framework improves software reusability, as the interfaces provided by a framework are defined as class attributes which can be applied to build new applications. Actually, the reuse of framework takes advantage of the expertise and effort of experienced software developer to minimize the time spent by subsequent developers on the same problem in the domain. Framework reuse not only improves software productivity, but also enhances the reliability and stability of software.
C. Extendibility
  Some frameworks provide hook methods allowing applications to extend their fixed interfaces, so that the extendibility is improved.

### Machine learning

Machine learning aims at simulating human activities using computers, so it is able to recognize known knowledge, gain new knowledge with which to improve its performance and optimize itself. Machine learning is being applied to various fields, such as biology [35, 36], economics [37, 38], chemistry [39, 40], and computer science [41, 42]. In 2020, Cunningham et al. applied machine learning approaches to prediction of signal peptides and other protein sorting signals [43]. In 2018, Azim et al. proposed a method for identifying emotions based on text using machine learning [44]. Companies, such as Amazon and IBM do research on machine learning as well. Amazon held a machine learning contest to verify whether it was possible to grant and revoke access to employees automatically. Researchers from IBM developed a system for disease inference by extracting symptoms from medical transcripts using machine learning techniques [45].

Generally, machine learning targets four categories of problems, which are regression, classification, clustering, and modeling uncertainty, known as inference.

A. Classification
  In classification, input data is divided into different categories. Normally, a classification task belongs to supervised learning, as the categories are labeled. The learning system gains knowledge, with which to assign new input data to one or more of these categories.
B. Regression
  To some extent, a regression problem is similar to classification, as it is also processed in a supervised way. The most significant difference is the output generated from regression problem is continuous, instead of discrete, like classification.
C. Clustering
  Clustering can be regarded as unsupervised version of classification. The basic functionality is also to classify a set of input into different classes; however, in clustering, the categories are not labeled anymore, which means the categories are generated as the system runs.
D. Modeling uncertainty
  Modeling uncertainty is not just to predict the frequency of random events. It integrates various factors that affect the occurrence of the event and analyzes the event using mathematical approaches, like Bayesian representation.

The process of establishing a face recognition system is to teach computers to mimic humans in recognizing human faces, i.e. a learning procedure. Therefore, machine learning becomes a perfect solution to this problem. In fact, machine learning approaches are frequently used in face recognition applications [46]. In Wang et al.’s work, a machine learning algorithm, Convolutional Neural Network, is combined with different classification techniques (decision tree, random forest), to build a system for facial expression recognition. The result suggests a mean accuracy of 93.85% across different datasets, and the system is able to operate in real-time [47].

## Framework for PCA-based face recognition

In this section, the classical PCA-based face recognition process is presented first, which shows the entire workflow and suggests some common approaches to the process. Then, a software framework for PCA-based face recognition system is proposed. All components contained in the framework are demonstrated in detail.

### General requirements

The framework’s target is to provide users with a tool, which is able to help them apply PCA to face recognition applications. Meanwhile, various extreme conditions, such as non-uniform illumination, shooting angle, and facial expressions need to be considered.

The first requirement of the framework is to describe the complete PCA-based face recognition system so that, software developers can use it as a guide to customize their own applications. Therefore, the framework intends to cover as many cases as possible.

Second, the framework needs to be flexible. Hence, each phase in the process needs to include multiple variations in order to deal with different situations. Moreover, the attribute of each variation should be described explicitly, thus making it easier for developers to select. We also mention possible combinations between different variations for developers’ reference.

Third, the model should be extendable. Since face recognition is still developing rapidly, more advanced techniques will be proposed to enhance the performance of current systems. The architecture of the framework should allow adjustment or enhancement in the future.

### PCA based face recognition process

**Fig 3:**
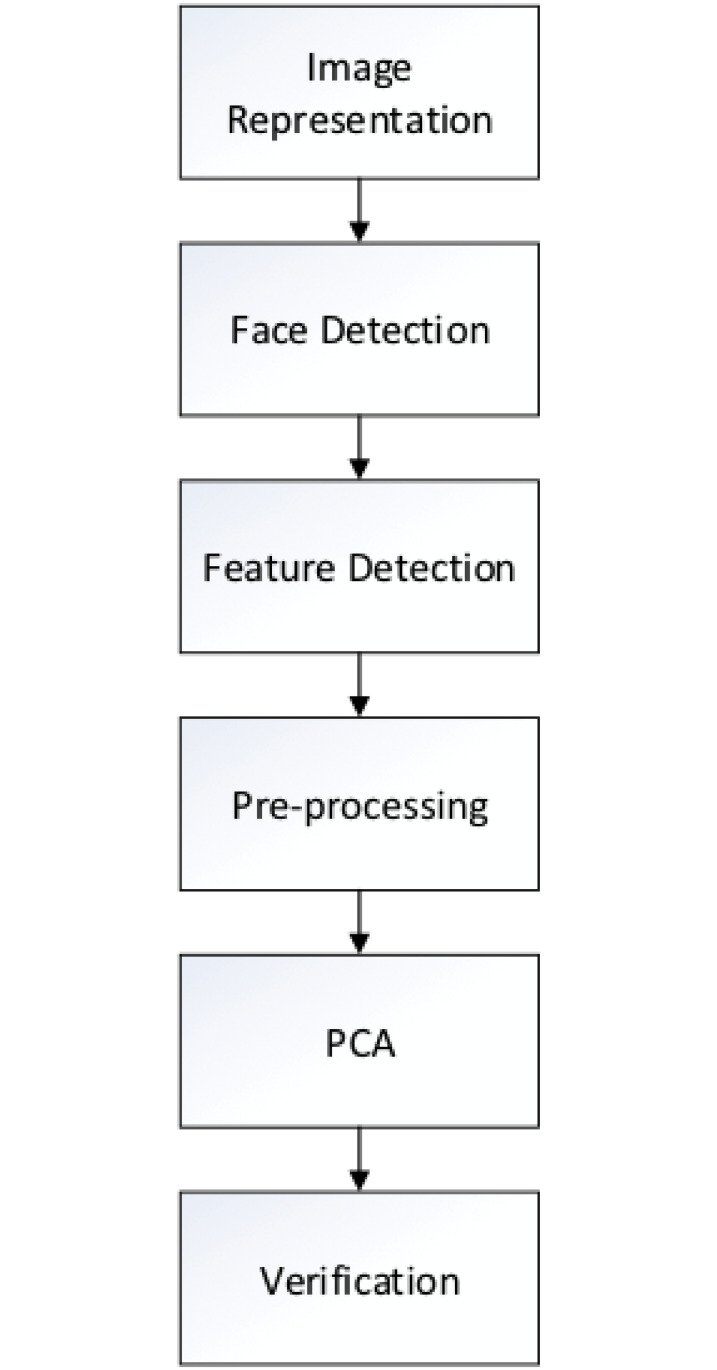
PCA-based face recognition system flow.

Fig 3 shows the entire facial recognition process with PCA in and includes six main steps: (1) image representation, (2) detecting face regions, (3) detecting facial features, (4) pre-processing, (5) conducting PCA, and (6) verification. Image representation is the step during which the image data is converted to a proper format. Face region detection and facial feature detection act on meta-data, preparing it for the following steps. Pre-processing is a step during which environmental influence, such as illumination, is reduced, so that the exact image information can be exhibited. Lastly, when Conducting PCA, thresholds defined at the verification step are used in image classification.

Face image verification, when using PCA, requires two image datasets: a training one, and a testing one. The former dataset provides data so that a customized PCA model can be built. The latter dataset contains images for verification.

#### Image representation

Usually, images are stored in a computer in a two-dimensional (2D) matrix format. Elements of this matrix represents pixels with values ranging from zero to 255. Color images have 3 different channels that are used to represent colors, such as red, green, and blue. An extra channel, named *α* is used to represent image transparency. The size of the matrix, its number of rows and columns, depends on image resolution. As a consequence, higher resolution images take more space for storage. Moreover, the size of matrix significantly affects matrix computation speed, creating a need for data compression methods. This motivates our use of PCA.

Image representation relates to more than just minimizing the image size. The selection of an appropriate image representation approach for the recognition algorithm improves efficiency and accuracy. This is discussed in more detail in Seciton.

#### Face detection

In face detection, a region containing a face is extracted from the background of the image. This technique is widely used in most smartphones of today and performs well in most situations. When detecting faces using smartphone cameras, an approximate face area may be good enough, however, it is important to note that when conducting face recognition, slight noise impacts the final result. Our framework depends on the chosen face detection technique, which then depends on the quality of the image containing a face to produce an accurate result. In some situations, such as when skin color is similar to background color, when part of the face is in shadow, or when the person is not looking straight to the camera, obtaining the face area is more challenging.

#### Feature detection

Image alignment is performed to achieve high recognition accuracy when using PCA. Usually, an affine transformation is preferred because of its simplicity and computation speed. To perform an affine transformation, three feature points on the face image are required. One common choice for these three points is the pupils of the eyes and the center point of the mouth. Thus, the main task of this step is to identify these three feature points in the face image.

**Fig 4:**
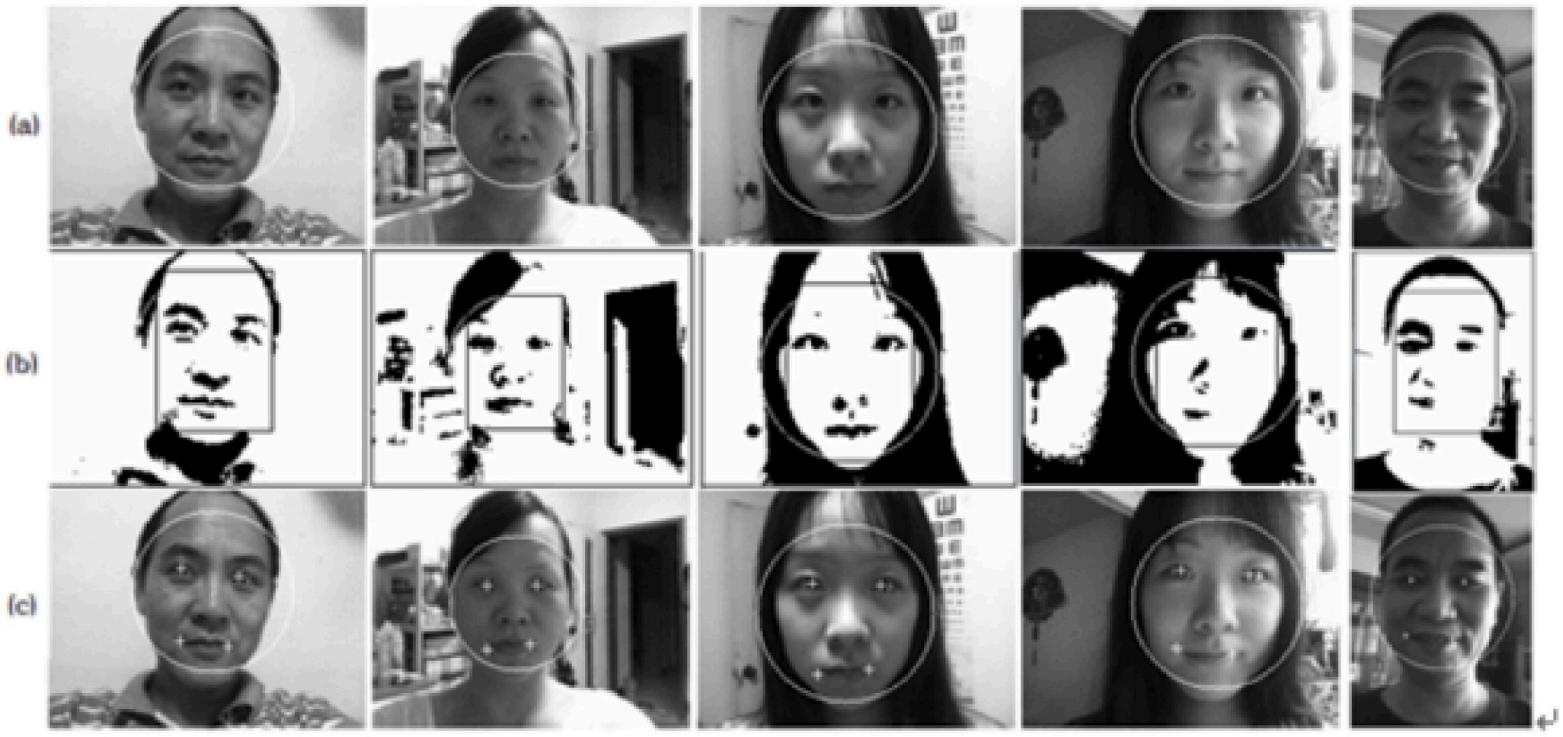
Feature detection [49].

In 2003, Peng et al., proposed a feature detection method based on weight similarity [48]. Initially, the approach transforms the image being analyzed into a binary format and the face area can be represented by *B*(*x*, *y*). Eq (9) shows the threshold for the binary image where *H*(*i*) stands for the histogram of the original image. Based on the pixel distribution of the face image, approximate areas for the left and the right eyes can be measured. These can be represented as *L*(*x*, *y*) and *R*(*x*, *y*), respectively. Since the color of pupils differs from other part of eyes, once a point *P*_*l*_(*x*, *y*) = 1 is found, it can be assumed as left pupil candidate. Similarly, once a point *P*_*r*_(*x*, *y*) = 1 is found, it can be assumed as right pupil candidate. If both of *P*_*l*_ and *P*_*r*_ meet the condition shown in Eq (10), they can be confirmed as the center points of two pupils. Note that, in the Eq (10), *γ*(*P*_*l*_, *P*_*r*_) is the similarity of the neighborhood of *P*_*l*_ and *P*_*r*_, and *D*(*P*_*l*_, *P*_*r*_) and *A*(*P*_*l*_, *P*_*r*_) are the distance constraint and angle constraint of *P*_*l*_ and *P*_*r*_ respectively. After identifying two pupils, the center point of the mouth *P*_*m*_ can be confirmed by integral projection. Fig 4 shows the flow of feature detection.

$$\begin{array}{c} B\left(x,y\right)=\{\begin{array}{cc} \begin{array}{cc} 0 & \;\text{if}\;A(x,y)\geq \theta \\ 1 & \;\text{if}\;A(x,y)\leq \theta \end{array} & ,\sum_{i=0}^{\theta }H\left(i\right)=15\%\times \sum_{255}^{i=0}H\left(i\right) \end{array}\} \end{array}$$ (9)

$$\begin{array}{c} B\left(x,y\right)=max(\gamma \left(P_{l},P_{r}\right)D\left(P_{l},P_{r}\right)A\left(P_{l},P_{r}\right)) \end{array}$$ (10)

#### Pre-processing

Image pre-processing is an important step in face recognition, since it is in this step that most factors that potentioally affect face recognition can be eliminated. There exists many different methdos to reduce noise, including histogram normalization or converting an image to a binary representation. Noise reduction is the goal of most of these methods, but some of them also change image format, which can then be used in later steps. This section continues the description of feature detection and introduces the process of affine transformation in images.

As previously discussed, three feature points, i.e., two pupils and the center point of the mouth, can be represented as *P*_*l*_, *P*_*r*_, and *P*_*m*_. An affine transformation aligns images according to the same template. These three feature points remain in the same position, and the other pixes are moved. Eq (11) describes the main idea of an affine transformation. Note that (*x*, *y*) stand for the pixels on the original face image and (*x*′, *y*′) is their resulting location in the template image after the transformation.

$$\begin{array}{c} {}[\begin{array}{c} x^{\prime }\\ y^{\prime } \end{array}]=[\begin{array}{ccc} a_{11} & a_{12} & a_{13}\\ a_{21} & a_{22} & a_{23} \end{array}][\begin{array}{c} x\\ y\\ 1 \end{array}],[\begin{array}{cc} a_{11} & a_{12}\\ a_{21} & a_{22} \end{array}]\neq 0 \end{array}$$ (11)

#### Principal component analysis

Karl Pearson invented the concepts of PCA in 1901 [11]. In the original idea, an orthogonal transformation is used to convert a set of observations of possibly correlated variables into a set of values of linearly uncorrelated variables called principal components. Years later, in 1991, Pentland and Turk introduced PCA to face recognition and proposed a new method called eigenface, frequently used in current face recognition research. Its basic idea is to extract the most significant information from face images and create a sub-space called feature space. The dimensionality of the feature space should be much smaller than that of the orignal images, but components used in face detection are preserved. The image set used to build this sub-space is called a training set, and the image set reflecting the components in the sub-space is called an eigenface. After creating this sub-space, a verification step is perfomed by projecting a testing image onto the space, generating a new image, and checking the similarity between this new image and the original one.

In Eqs (12) and (13), *M* stands for the dimensionality of the feature sub-space, *U*_*k*_, *k* = 1, 2, …, *M* are the eigenfaces, *ω* stands for the average face.

**Fig 5:**
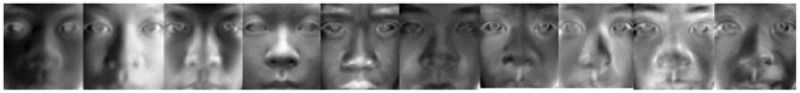
Eigenfaces [49].

Fig 5 shows a set of eigenfaces generated from a training dataset that contains about 200 images.

**Fig 6:**
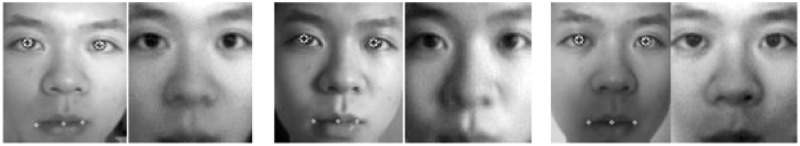
Original images and projected images [49].

Fig 6 shows 3 pairs of images containing the original one and the image created after being projected to the sub-space. As the images of the training dataset are of the same person, so the projected images are relatively similar to the original ones.

$$\begin{array}{c} X^{\prime }=\sum_{k=0}^{M}\omega_{k}U_{k},\;k=1,2,\ldots ,M \end{array}$$ (12)

$$\begin{array}{c} \omega_{k}=U_{k}^{T}\left(X-\varphi \right),\;k=1,2,\ldots ,M \end{array}$$ (13)

#### Verification

In the verifiation step, the original input image is compared with its projection of the feature sub-space. There are many approaches for similarity calculation and the proper approach choice may lead to better results.

As explained, images are represented as a matrix. This means that verification becomes a task of comparing the similarity of two matrices or vectors. This can be done with statisical methods.

Popular distance measures such as Euclidean distance, Manhattan distance, Chebyshev distance, and Minkowski distance are good choices for this task. Each distance measure has advantages and disadvantages, so choosign a suitable measure for the problem is important.

### Framework model

**Fig 7:**
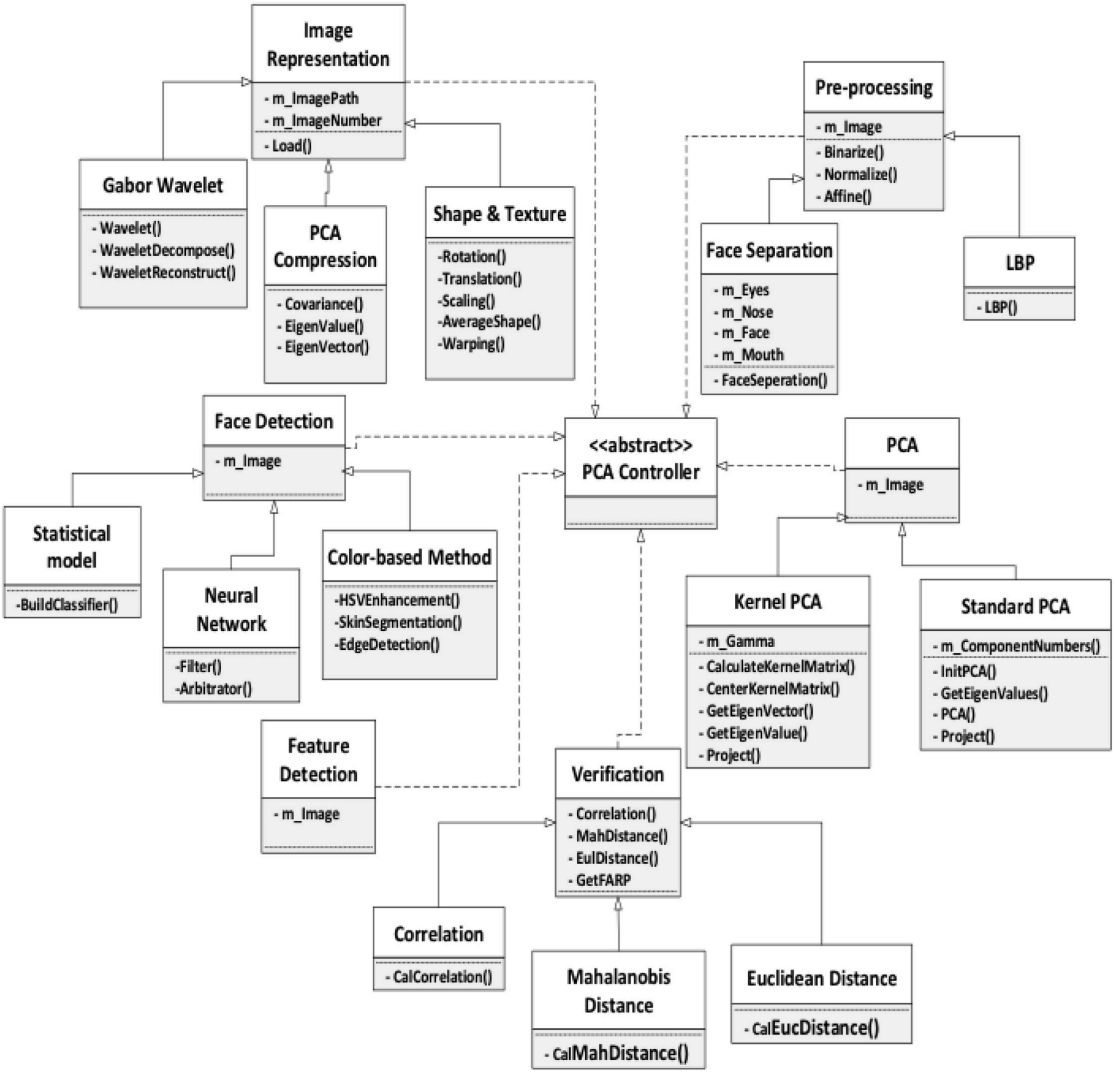
Framework design model.

In this seciton, we introduce and discuss a face recognition system with PCA (Fig 7). The framework describes the whole face recognition process, and for each phase in the process, some possible variations are presented, so that face recognition approaches can be adapted to different cases and software developers are assisted when customizing their applications. The framework takes into consideration face recognition in extreme situations, such as non-uniform illumination, exaggerated facial expression, shooting angle of the images, and the image data type. In addition to the options included in the framework, we also suggest other potential appraoches. The framework is outlined below.

For the face representation step, we will discuss the the approaches Gabor wavelet, PCA expression, and shape and texture expression. In addition, for the face detection step, we will talk about statistical model, neural networks, and color based methods. For pre-processing, we consider face separation, and local binary pattern (LBP). Lastly, for the PCA step, which is the core step, we will talk about Kernel PCA and standard PCA.

**Fig 8:**
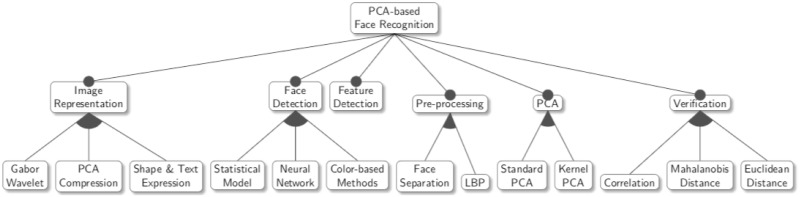
Framework as a feature diagram.

The framework can be represented as a feature diagram, as in Fig 8, where each one of the steps of the process assumes a different technique. The choice of features yields different variations for each step of the face recognition process, each with its own benefit and suitable situation. The combination of these variations allows the framework to provide a minimum of 108 application instances: three variations for the face recognition, face detection, and verification steps; and two variations for the pre-processing and PCA steps. However, the actual number of possible application instances that can be captured by our framework is significantly higher than 108, since in reality, some variations can be combined (when in the same step), be omitted, or collaborate with other simple mathematical operations. As a result, a conservative estimate of the number of application instances captured by our framework exceeds 150. Although these instances do not cover all possible situations for face recognition, our framework provides a great help to software developers.

#### Image representation

**Fig 9:**
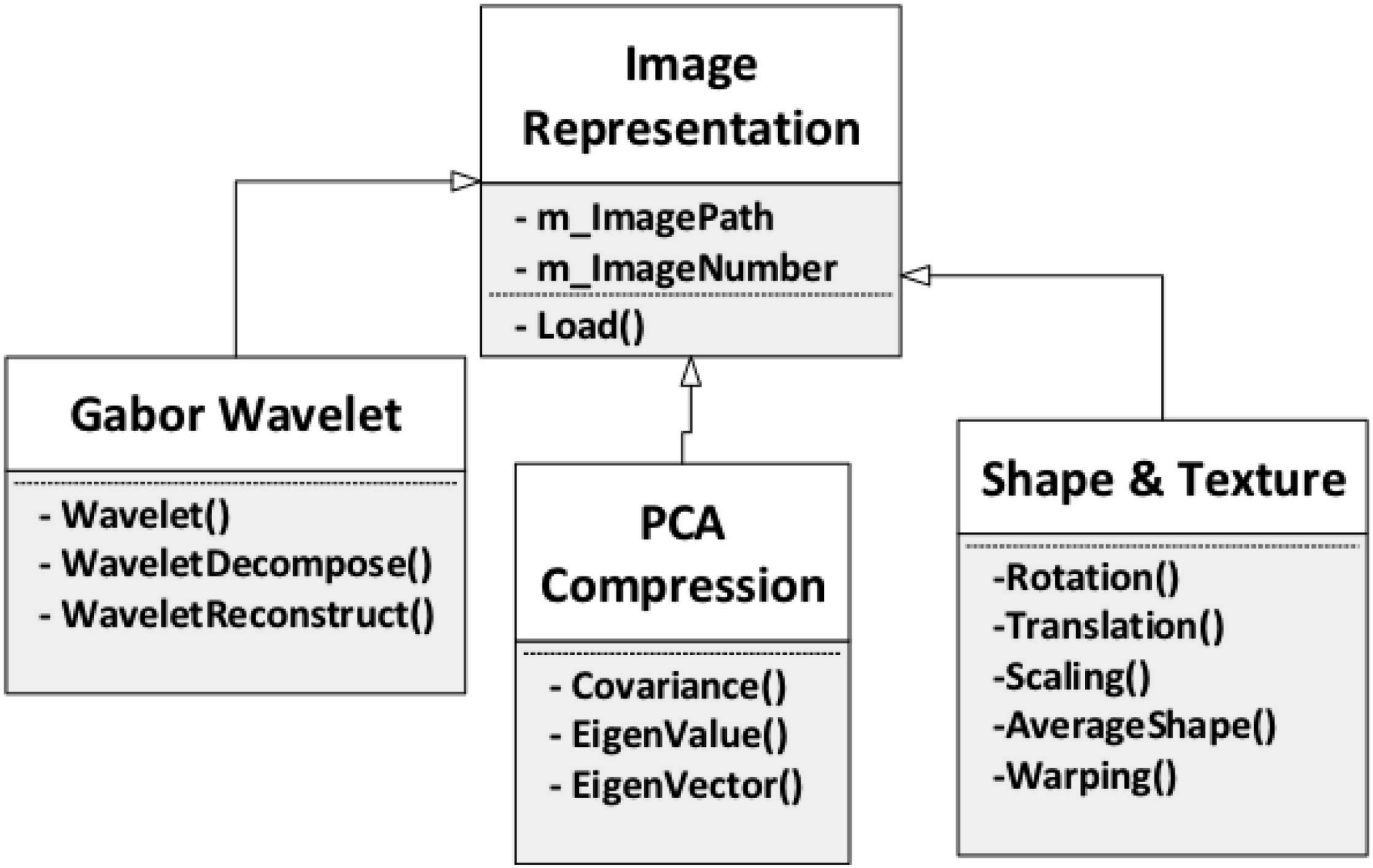
Image representation.

As the first processing step of face recognition, image representation plays an important role not only in explicitly representing the image information but also in reducing noise and compressing data. Appropriate selection of image representation approaches facilitates the later steps and improves the overall performance of the entire system. In this section, we present three different variations for representing images, which are Gabor Wavelet, PCA Compression, and Shape and Texture, as shown in Fig 9.

*Gabor wavelet*. Image processing methods are mainly divided into two categories, which are spatial domain analysis and frequency domain analysis. Spatial domain analysis directly processes the image matrix; however, frequency domain approaches convert the image from the spatial domain to the frequency domain, and then analyze the image feature from another perspective. Spatial domain analysis is widely used in image reinforcement, image reconstruction, and image compression [44].

Fourier transforms are one of the earliest method of transferring signals from the spatial domain to the frequency domain, as shown in Eq (14). After processing the signal, an inverse transform can transfer the signal back to spatial domain through Eq (15).

$$\begin{array}{c} F\left(\omega \right)=\int_{-\inf}^{\inf}f\left(t\right)e^{-j\omega t}dt=F\left[f\left(t\right)\right] \end{array}$$ (14)

$$\begin{array}{c} f\left(t\right)=\frac{1}{2\pi }\int_{-\inf}^{\inf}F\left(\omega \right)e^{-j\omega t}d\omega =F^{-1}\left[f\left(t\right)\right] \end{array}$$ (15)

Classic Fourier transform provides a powerful tool for image processing; however, it is only able to reflect the integral attributes of signals, which means it lacks the ability to do local analysis.

Based on Fourier transform, Dennis Gabor proposed a new transform which only depends on part of the signal and is able to extract local information [50].

The basic idea of the Gabor transform is to divide the signal into multiple intervals, and then analyze each interval using a Fourier transform, so that the frequency in certain interval can be obtained. In image processing, a Gabor transform is also known as Gabor wavelet.

A Gabor wavelet is similar to the stimulation of a simple cell in a human being’s visual system [51]. It captures salient visual properties such as spatial localization, orientation selectivity, and spatial frequency. Furthermore, as Gabor wavelet is insensitive to illumination variation, it provides good adaptability to illumination variation in image representation.

Gabor wavelet is particularly suitable for image decomposition and representation when the goal is the derivation of local and discriminating features. In 1999, Donate et al. [52] showed that the Gabor filter representation had better performance for classifying facial actions. In 2003, Liu et al. [53] presented an independent Gabor feature method for face recognition. The method achieved 98.5% correct face recognition accuracy on FERET dataset, and 100% accuracy on ORL dataset.

*PCA compression*. As the core of this study, PCA is the main step of a face recognition system that we discuss. However, it also can be used as an image representation method when combining with other recognizing approaches. When representing face image using PCA, the main idea is to transfer the original image to a format with lower dimensions, i.e., to represent by a smaller number of parameters.

PCA was first applied to the realm of pattern recognition in 1965 by Watanabe [54]. In 1990, Kirby et al. introduced the method to face recognition, particularly characterization of human faces [55]. The work introduces a concept of optimal coordinate system, in which the set of basis vectors which makes up the system are referred to as eigen-pictures. They are actually the eigenfunctions of the covariance matrix of the ensemble of faces. For the evaluation of the procedure, face images from outside of the dataset are projected on the set of optimal basis vectors. The result shows a 3.68% error rate out of over ten face images, which dominated in this field at the time.

As the development of face recognition technique evolves, some variations of PCA-based image representation method have been invented. Moreover, PCA-based image representation is always combined with other recognition approaches to achieve better recognition accuracy.

In 2005, Zhang et al. proposed a two-directional two-dimensional PCA for face representation and recognition [56]. It has been proved that 2DPCA outperforms standard PCA in terms of recognition accuracy. However, 2DPCA needs more coefficients for image presentation than PCA. Therefore, Daoqiang and Zhi-Hua conduct PCA on row and column directions simultaneously, which results in a same or even higher recognition accuracy than 2DPCA, though with less coefficients needed.

*Shape and texture expression*. Shape and texture expression methods represent human faces using geometric features. Since face color or illumination are not considered in shape and texture expressions calculations, the amount of noise that impacting the recognition accuracy is reduced. In fact, shape-based and texture-based methods were independent initially. After proving that the shape-based method cannot perfectly solve the problem caused by expression, scale, and illumination, texture is introduced to be combined with shape to achieve better recognition accuracy [56].

Liu et al.’s paper in 2001 [57] clearly explains the work flow of shape and texture-based face expression methods. We use some of the figures and explanations to demonstrate the principle in detail.

The shape of face images reflects the contours of face, so a set of control points derived by manual annotation are used to describe the contour. To underscore the shape features of face, these points ignore other facial information like color, gray scale. They only depict feature points such as eyebrows, eyes, nose mouth, and the contour of face. Generally, the shape image is generated from a large set of training images. After obtaining the shape from each training image, the shapes are aligned by rotation, translation, and scaling transformations.

First, calculate the average of the aligned shapes of training images to obtain the mean shape image. Then, warp the normalized (shape-free) face image to the mean shape to generate a new image, which is the texture. The warping transformation basically separated the image into multiple small triangular regions and then performed affine transformation on each of them to warp the original face to the mean shape. These two steps result in a texture (shape-free image) which has the same face contour as the mean shape.

The experimental result of Liu et al.’s work shows that the integrated shape and texture features capture the most discriminating information of a face, which contributes to their high recognizing accuracy. Besides their work, other research [58–60] demonstrate the advantages of using shape and texture method to represent facial image.

#### Face detection

**Fig 10:**
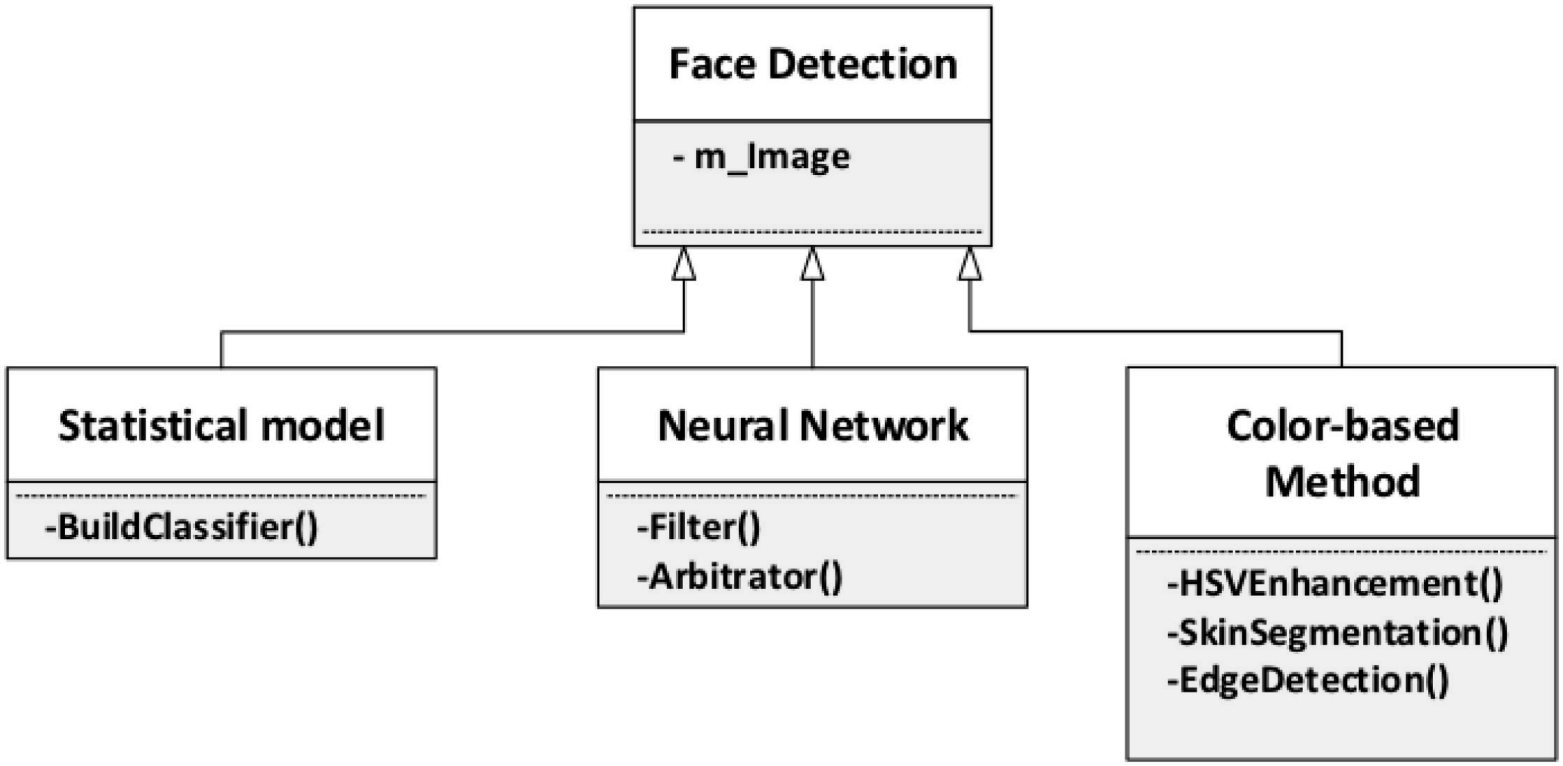
Face detection.

Face detection identifies the area in the image containing a face that will be used in the entire recognition process. The accuracy of this detection has great impact in the final result, since the presence of background noise does affect most face recognition algorithms. For this phase, we provide three variations, which are the statistical model, Neural Network, and color-based method, as shown in Fig 10.

*Statistical model*. The complexity of human face images makes the detection of face features to be difficult, therefore statistical-based detection methods have been attracting researchers’ attention. This method regards the face region as a type of pattern, also known as pattern feature, and uses a large number of face image and non-face image to train and generate a classifier. So the more training the detection method receives, the more robust it will be.

Feature space-based methods, such as PCA, LDA, probabilistic model-based methods, and support vector machine-based (SVM) methods all belong to statistics-based detection methods. Actually, neural networks-based methods, which are discussed next, also utilize statistical principles; however, we explain it individually because of some of its peculiarities.

In 2000, Schneiderman et al. [61] proposed a statistical method to detect 3D objects. The method uses the product of histograms and detects both object appearance and “non-object” appearance. Each histogram represents the joint statistics of a subset of wavelet coefficients and their position on the object. Many of such histograms are used by the approach and they represent a large variety of visual attributes. The result demonstrates detection accuracy.

In 1997, Moghaddam et al. proposed a probabilistic visual learning for object representation, which is based on density estimation in high-dimensional spaces using an eigenspace decomposition [62]. The technique has been applied to not only face detection, but gesture recognition.

*Artificial neural network*. An Artificial Neural Network is a computation model consisting of many neurons, in which each neuron includes a specific output function called an activation function. The connection between every two neurons has a weight that processes the output from the first neuron.

The most significant attributes of artificial neural networks are their adaptability and parallelism. Adaptability grants its ability to learn through training and autonomously correct weights in connections to avoid the same faults. As each neuron in the network is responsible for a certain job, an artificial neural network is able to work in parallell, which facilitates processing big data such as images.

Because of these two remarkable attributes, artifical neural networks have been attracting attention from researchers in face recognition. In 1997, Lin et al. proposed a face detection method using a probabilistic decision-based neural network [63]. The detection accuracy reaches 98.34%. In 1998, Rowley et al. [64] introduced a face detection system that identifies upright front faces using neural networks. First, part of the image, which might contain a face region, is obtained. Then a series of pre-processing steps, such as light correction and histogram normalization, are used with the goal of reducing noise. Finally, a neural network decides whether there exists a face. Results report an accuracy of face detection between 77.9% and 90.3% using set of 130 test images, with an acceptable number of false positives.

*Color-based methods*. Traditional face detection approaches are always performed in gray-scale space, in which the gray-scale is the only information that can be captured. Moreover, since there is no limit for area or proportion, it is necessary to search the entire space, which is fairly time-consuming. However, if color information can be introduced, the search area will be narrowed, because the skin color is the most straightforward information on a human face. In addition, in the face region, skin color dominates.

A problem that needs to be considered is the difference of skin color. Fortunately, research in this field shows that skin color in certain color space aggregates, especially when the illumination factor is removed [65]. Therefore, using skin color as a clue to exclude any area which is not skin can be easily performed.

When applied to face detection, skin color information is always used in three different phases of face detection. It could be used as the core function, the pre-processing method, or in post-verification. For instance, in 2002, Sahbi et al. proposed a skin color approach for face detection combined with image segmentation 2002. In their approach, the images are first separated coarsely to provide regions of homogeneous statistical color distribution. Then the color distribution will be used for training a neural network to detect faces. The experiment result shows an accuracy of around 90%.

#### Pre-processing

**Fig 11:**
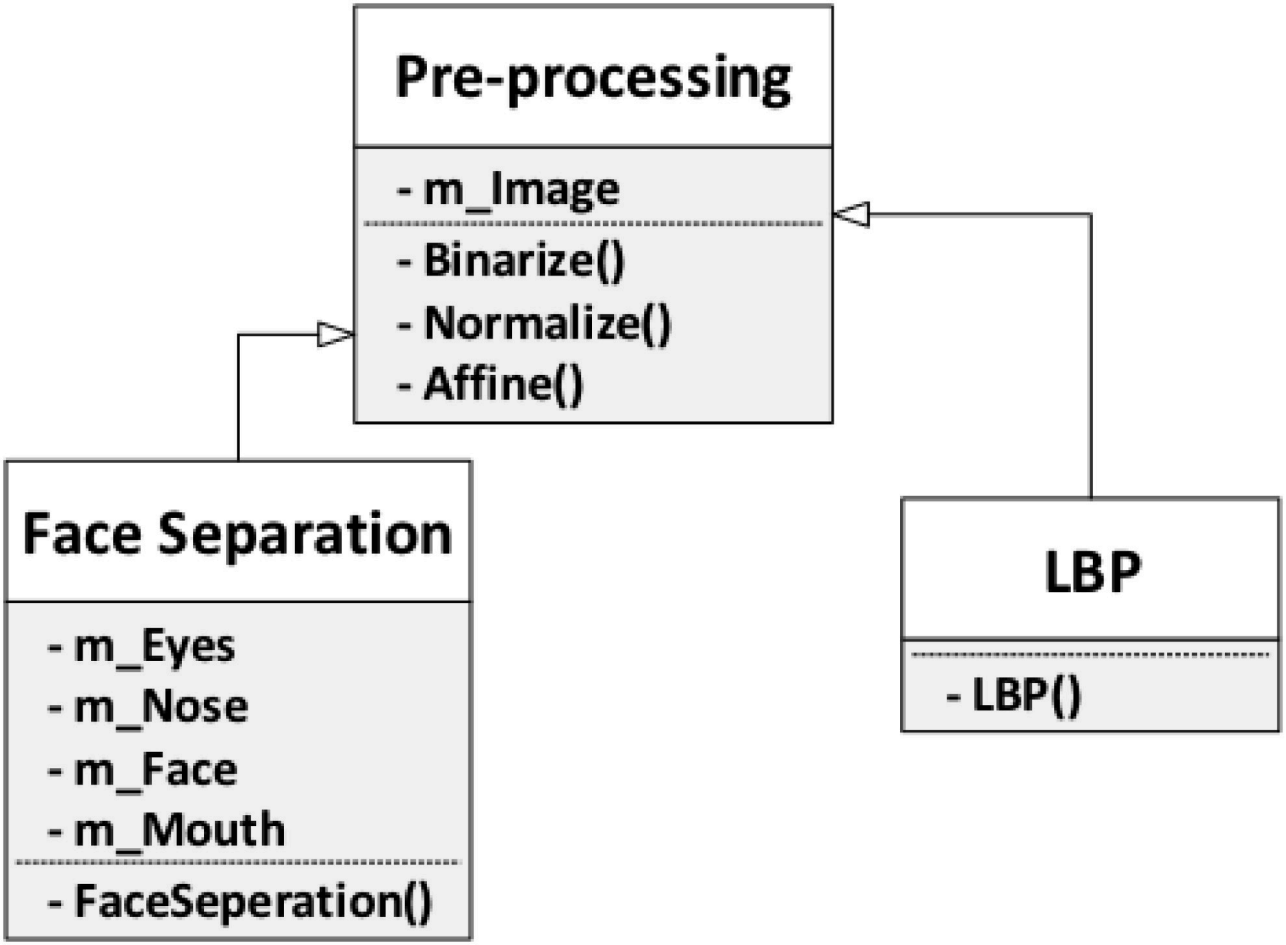
Pre-processing.

A pre-processing step can be regarded as a filter which reduces major noise impacting the following recognition process. It aims at generating clear images with useful information retained. This section presents two pre-processing approaches: face separation and LBP (Fig 11). The first handles face images with exaggerated expressions and the second deal with non-uniform illumination.

**Fig 12:**
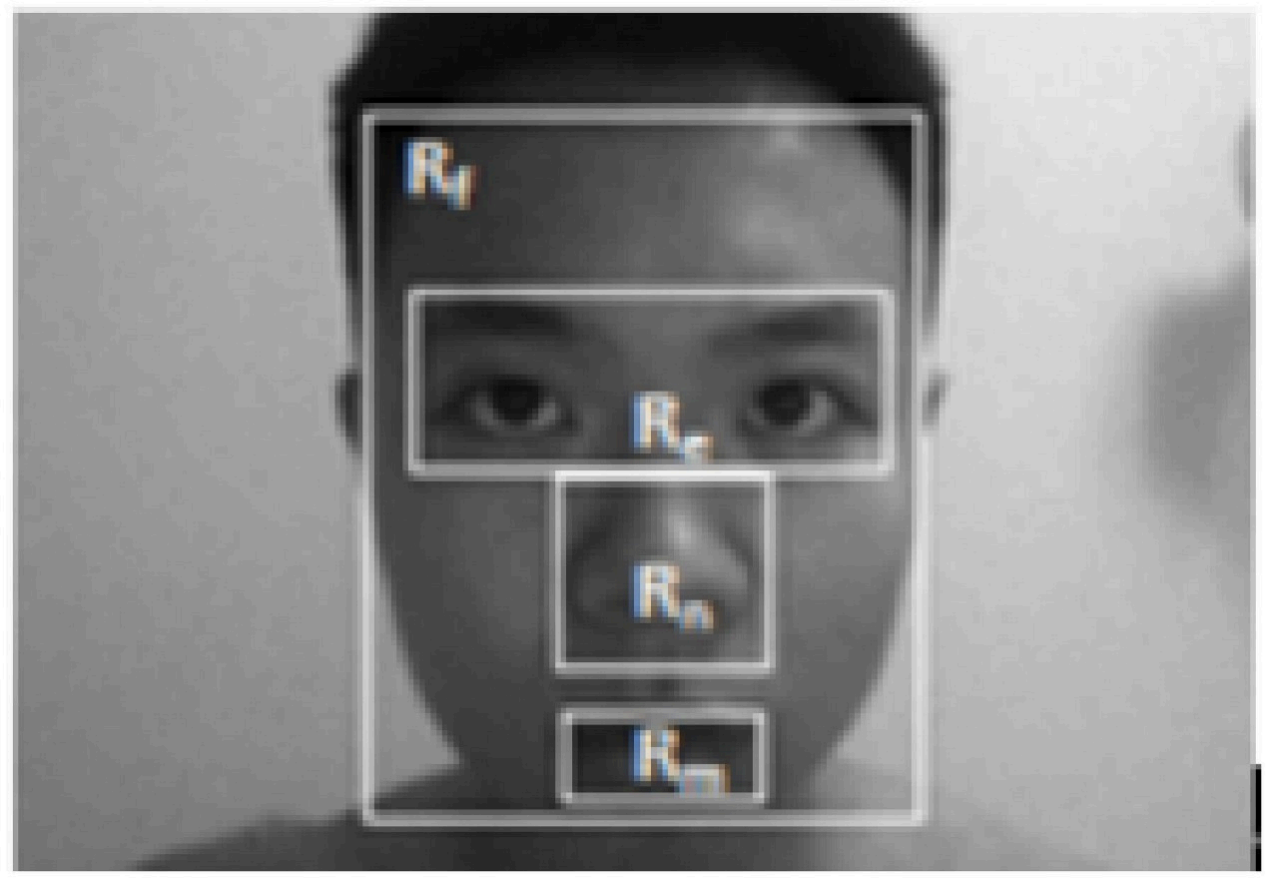
Face separation [49].

*Face separation*. When taking pictures, it is expected that people will have facial expressions, and a good face recognition system should be able to perform well under these conditions. However, most systems do not expect exaggerated facial expressions, and the presence of these expressions impact the recognition process, especially for feature detection and image aligning. To mitigate this problem, this pre-processing method divides a face image into four parts: eyes, nose, mouth, and the entire face. This allows the system to perform the following steps on each part, as shown in Fig 12.

In 2013, Peng et al. used face separation for standard PCA-based face recognition calculation [49]. They applied PCA on each part of the face mentioned previously, and integrated the results using Eq (16). Note that, in Eq (16) *δ*_*F*_ refers to the score of entire face, *δ*_*M*_ refers to the score of the mouth, *δ*_*N*_ refers to the score of the nose, and *δ*_*E*_ refers to the score of eyes. The weights assigned to each part is obtained from experimentation. The result shows significant progress in recognizing face images with exaggerated expressions.

$$\begin{array}{c} \delta =0.40\times \delta_{F}+0.10\times \delta_{M}+0.100\times \delta_{N}+0.40\times \delta_{E} \end{array}$$ (16)

**Fig 13:**
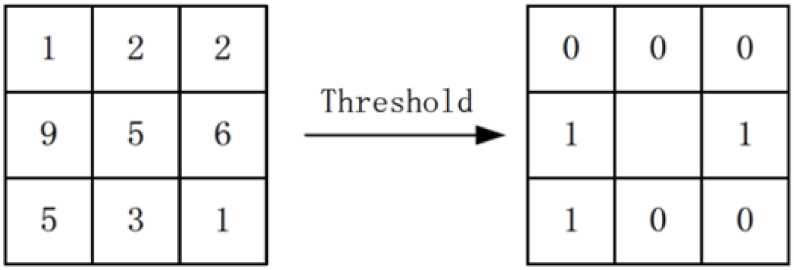
LBP.

**Fig 14:**
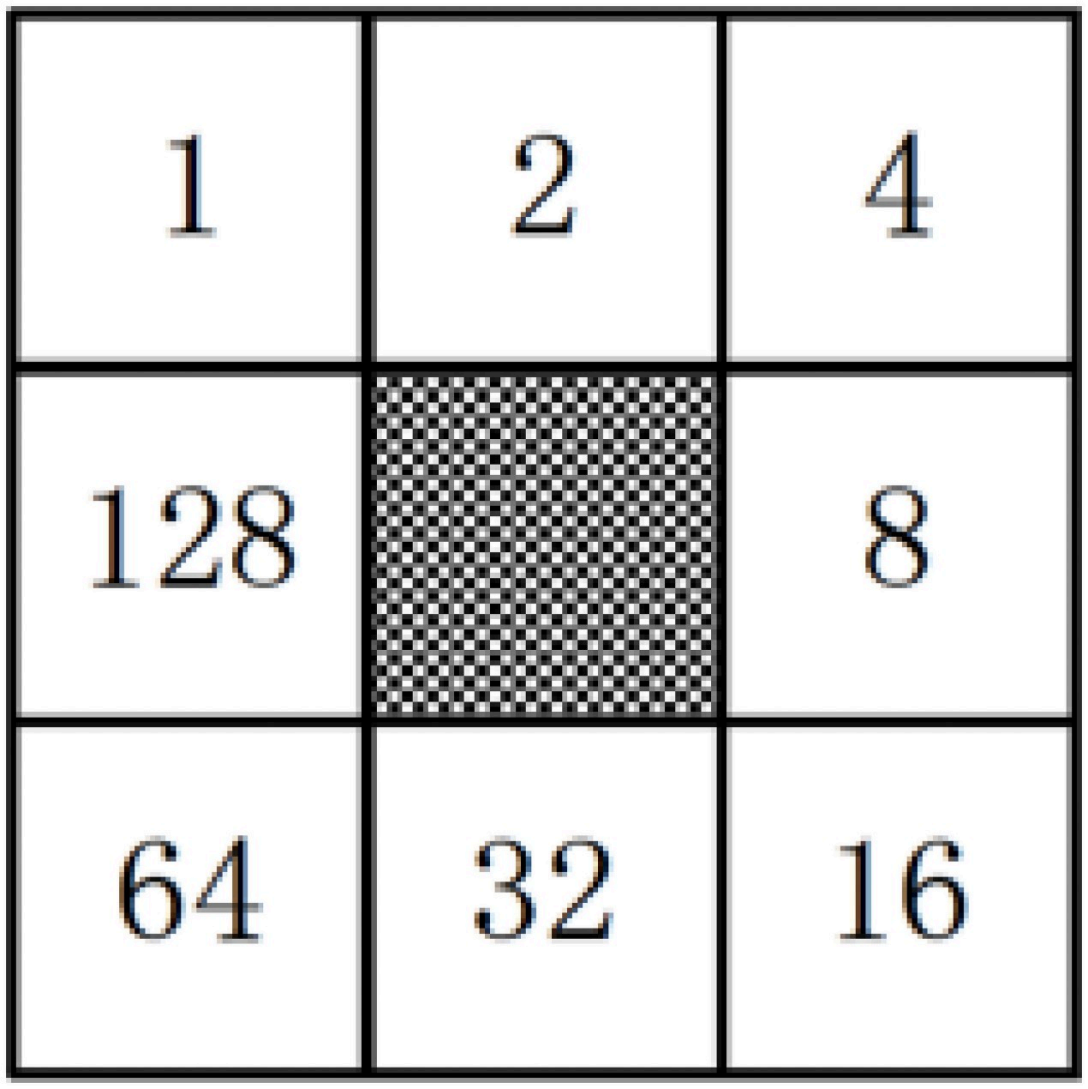
LBP weight.

**Fig 15:**
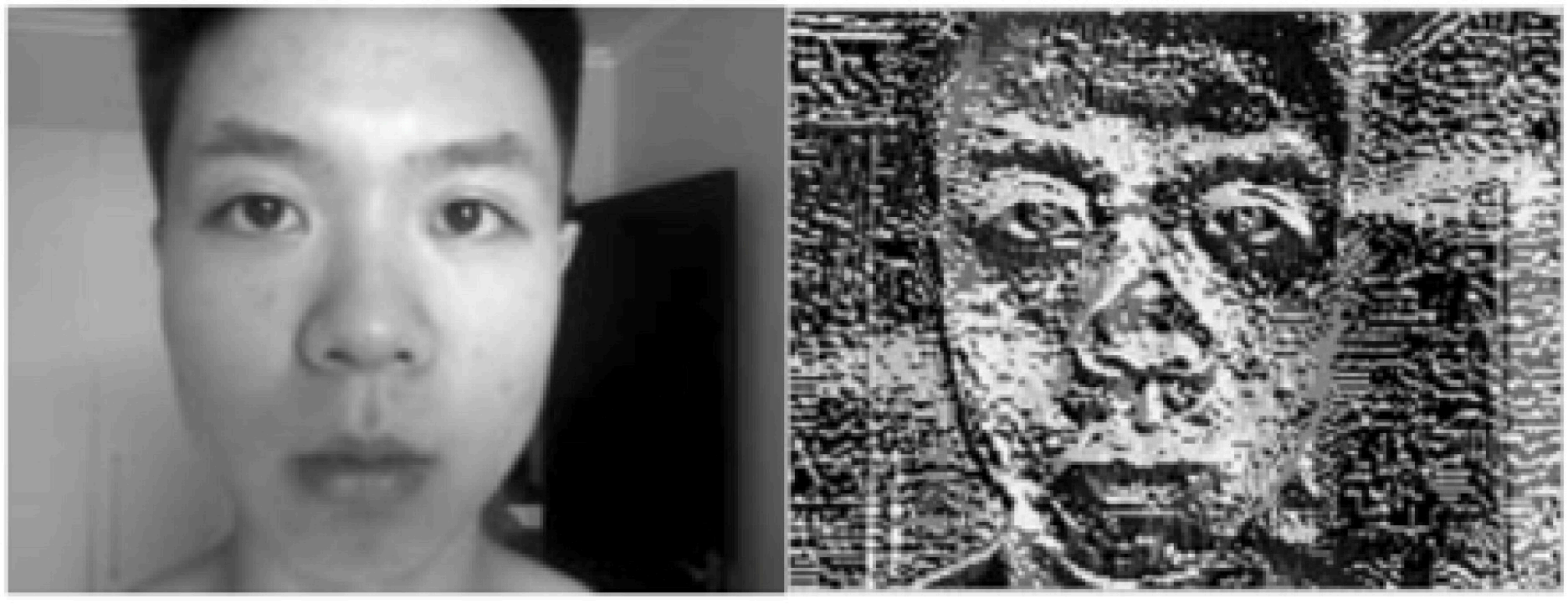
LBP result.

*Local binary pattern*. Local binary pattern (LBP) was introduced by He et al. in 1990 [66]. Put simply, its goal is to calculate a weighted sum for a single pixel with it neighboring pixels. Generally, the window size of sum is set as 3 × 3. It is still creating a binary representation of an image. LBP traverses the image using each pixel as a center point and performs a calculation for all of the eight neighboring pixles. If a pixel has a gray value less than the gray value of the center point, LBP assigns that pixel a value of zero; otherwise, it assigns a one, as shown in Eq (17) and Fig 13, where *I*(*Z*_*i*_) represents neighboring pixels, and *I*(*Z*_0_) represents the center pixel. Fig 13 gives an overview of the assignment process. The weights assigned to each pixel are always different. Fig 14 shows one possible weight distribution. Fig 15 shows an image which is processed by LBP.

$$\begin{array}{c} f\left(I\left(Z_{0}\right),I\left(Z_{i}\right)\right)=\{\begin{array}{cc} 0, & if(I\left(Z_{i}\right)-I\left(Z_{0}\right)>0)\\ 1, & if(I\left(Z_{i}\right)-I\left(Z_{0}\right)<0) \end{array},i=1,2,3,\ldots ,8 \end{array}$$ (17)

LBP usually performs well on image classification. Since the computation is relatively simple, it is also efficient for most cases. However, some limitations are worth mentioning such as low extendibility and scalability.

**Fig 16:**
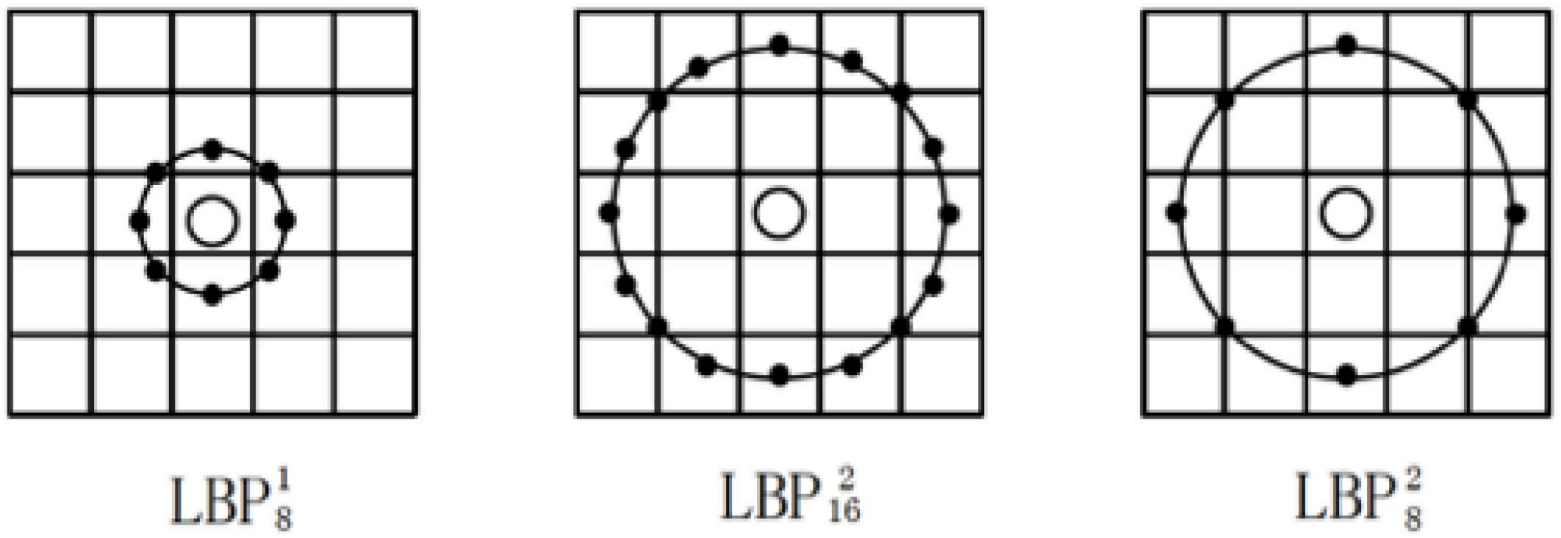
Circle LBP.

**Fig 17:**
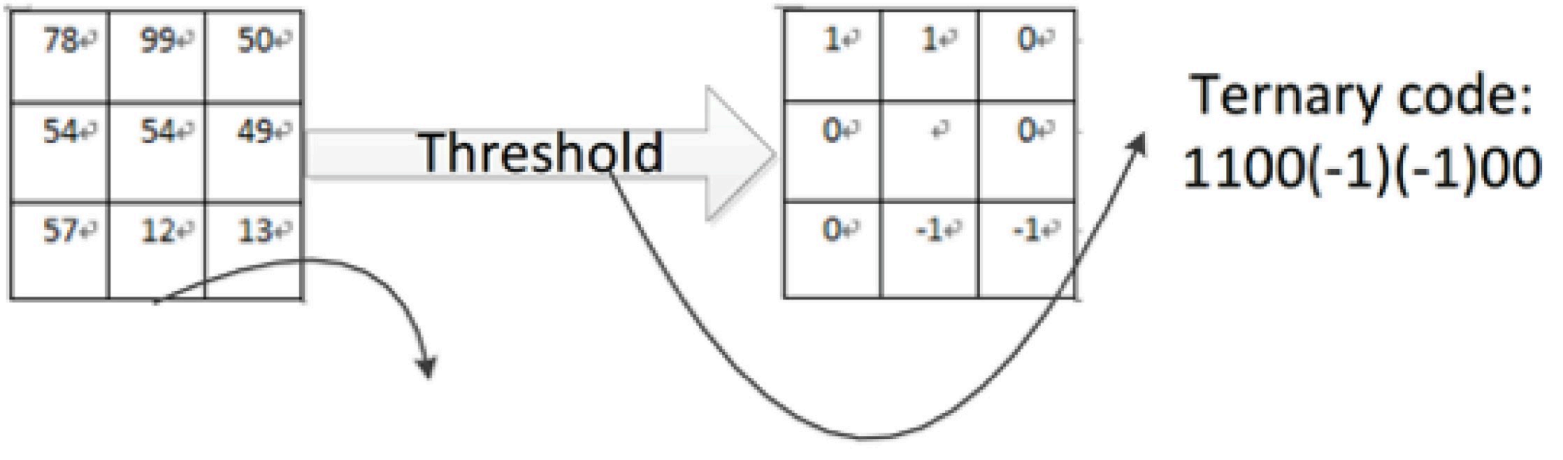
LTP.

After a decade of research, some variations of the LBP algorithm have partially overcome these limitations. In 2002, Ojala et al. [67], used a circular neighborhood with arbitrary radius instead of a 3 × 3 window (Fig 16). In 2010, Tan et al. [68], proposed Local Ternary Patterns (LTP), a LBP-based method that compares the values of neighboring pixels with the value of the center pixel plus a range value *t*. As a consequence, the eight neighbor values could be encoded. Fig 17 shows this process. Besides these variations, researchers also combined LBP with other algorithms, to enhance the efficiency.

#### PCA

**Fig 18:**
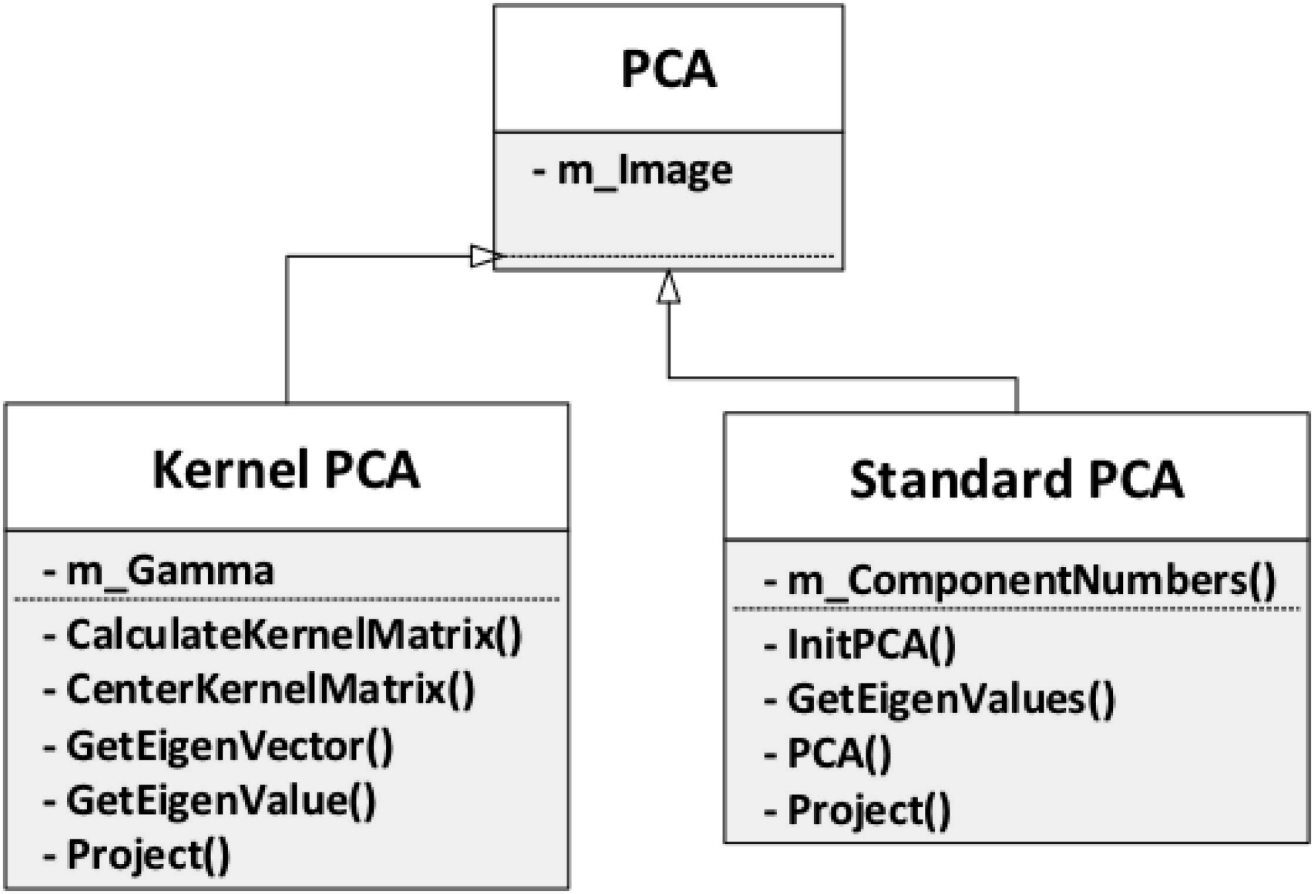
PCA.

In this section, we discuss the core step of the PCA-based face recognition framework: PCA. Two variations are provided and shown in Fig 18: Standard PCA, mainly used for linear image data, and kernel PCA, used for non-linear image data.

*Standard PCA*. Principal Component Analyiss (PCA) is generally used to reduce the dimensions of the dataset with minimal loss of information. Here, the entire dataset with *d* dimensions is projected onto a new subspace, with *k* dimensions, where *k* ≪ *d*.

The standard PCA approach can be summarized by the following six steps [69]:

1. Compute the covariance matrix related to the original d-dimensional dataset X.
2. Compute the eigenvectors and eigenvalues of the dataset.
3. Sort these eigenvalues by decreasing order.
4. Choose the k eigenvectors that correspond to the k largest eigenvalues where k is the number of dimensions of the new feature subspace.
5. Construct the projection matrix W from the k selected eigenvectors.
6. Transform the original dataset X to obtain the k-dimensional feature subspace Y.

**Fig 19:**
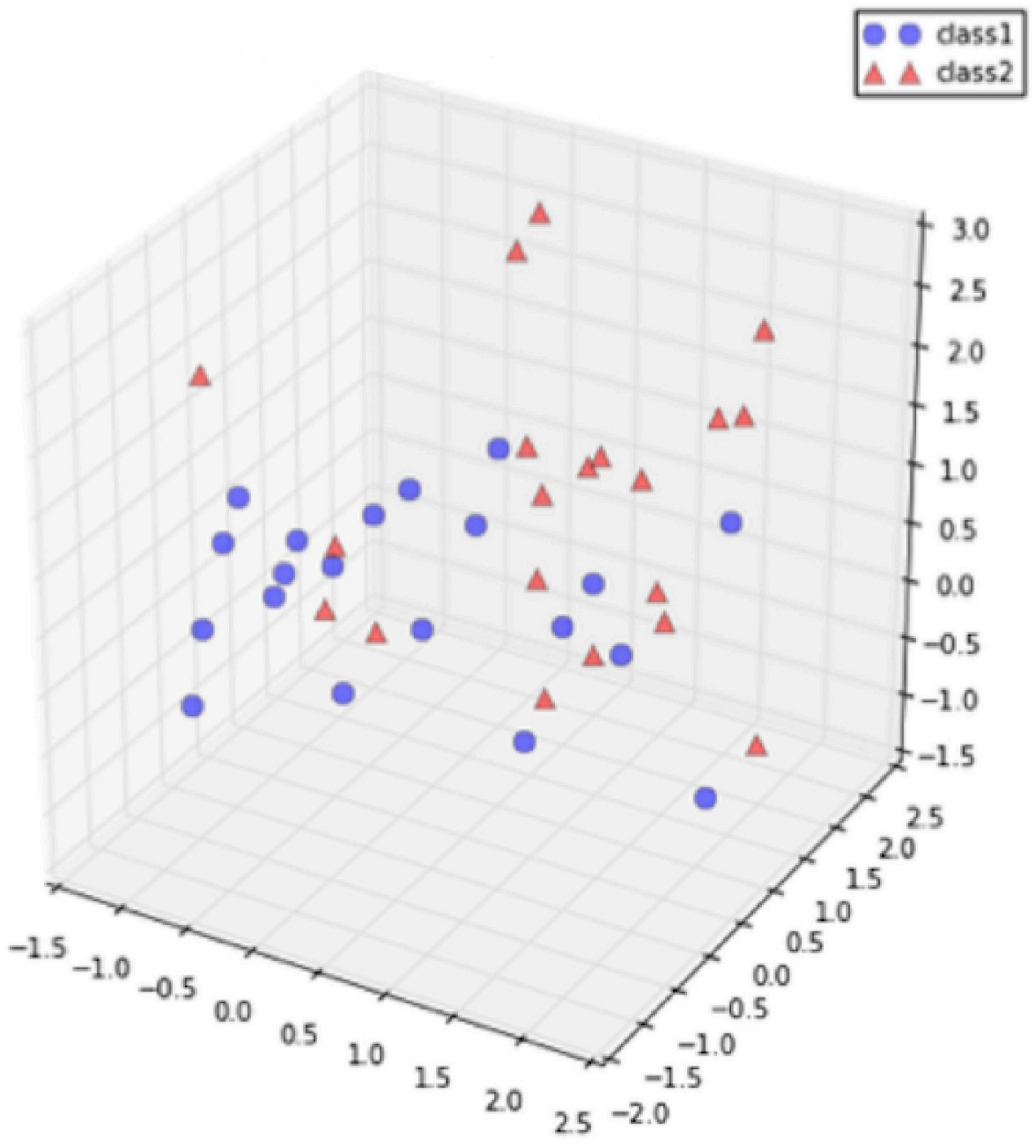
Original dataset [69].

**Fig 20:**
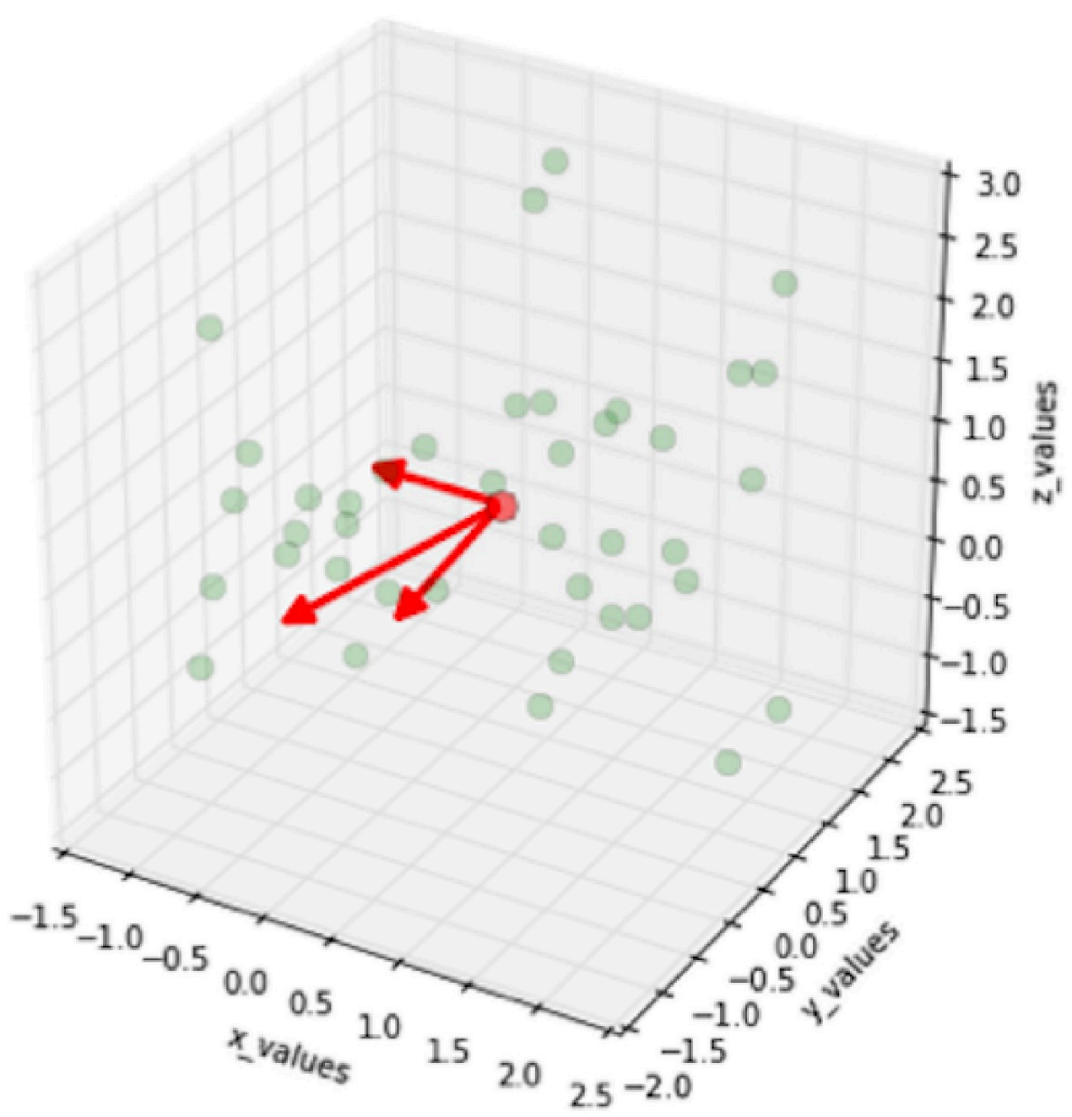
Eigenvalues and eigenvectors [69].

**Fig 21:**
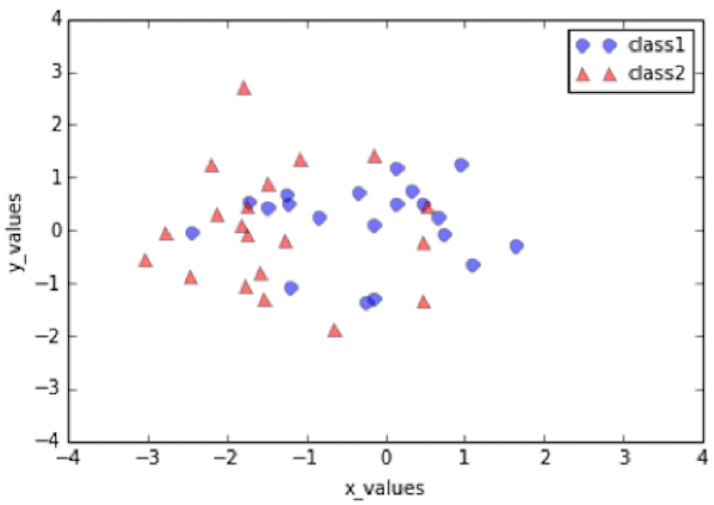
Classification by using standard PCA [69].

Figs 19–21 show an example of the use of PCA on a three dimensional dataset. Fig 19 shows the original dataset. Two categories of data are mixed together and hard to be classified. Fig 20 depicts the eigenvalues and eigenvectors of the original dataset. After being processed by PCA, the dimensionality is reduced to 2 and the classification is clearer, as shown in Fig 21.

Turk and Pentland first used PCA on face recognition in 1991 [30]. We have introduced the basic principles in previous sections. Although PCA has been researched for decades, many still prefers it for face recognition tasks because of its robustnes, extendibility, and ease to combine it with other existing methods.

*Kernel PCA*. Kernel PCA is an extension of the PCA algorithm that uses techniques of kernel methods. It starts with mapping the input space into a feature space via nonlinear mapping and then proceeds with a computation of the principal components in that feature space.

**Fig 22:**
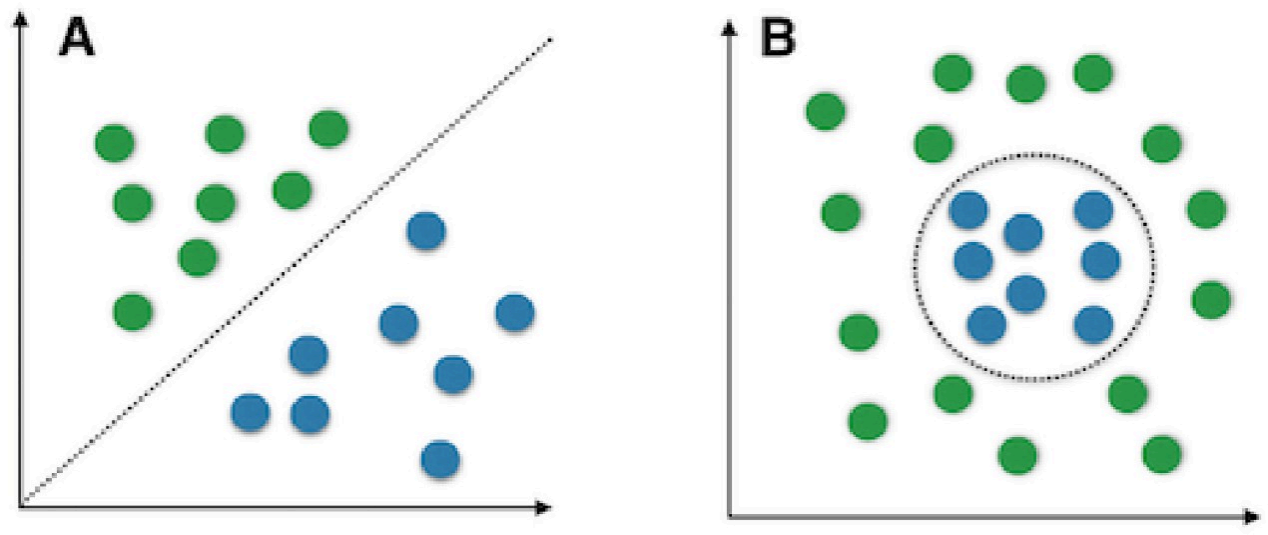
Linear (left) and nonlinear (right) data types [69].

Standard PCA works well when data is linearly separable. However, in practice, this is usually not the case because of the impact of external factors on image data, such as shooting angle, illumination, and other types of noise. This shows the need of a method that handles nonlinear cases. A comparison between linear data and non-linear data is shown in Fig 22.

The implementation of Kernel PCA has 2 main steps:

1. Compute the kernel (similarity) matrix.
2. Eigen decompose the kernel matrix.

**Fig 23:**
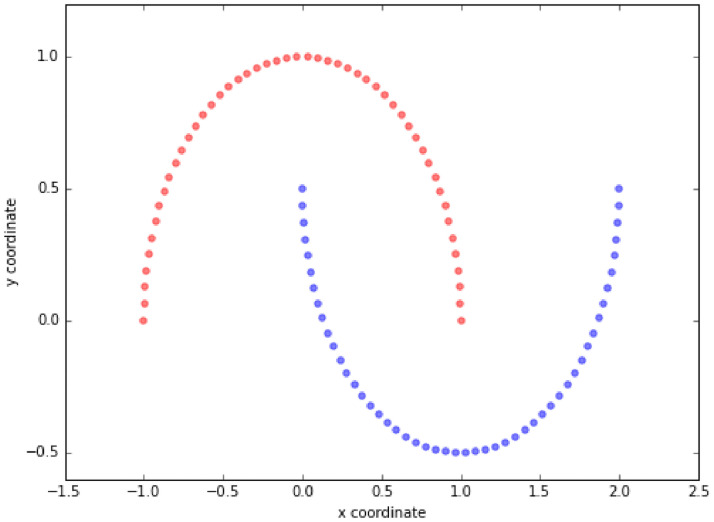
Original data [69].

**Fig 24:**
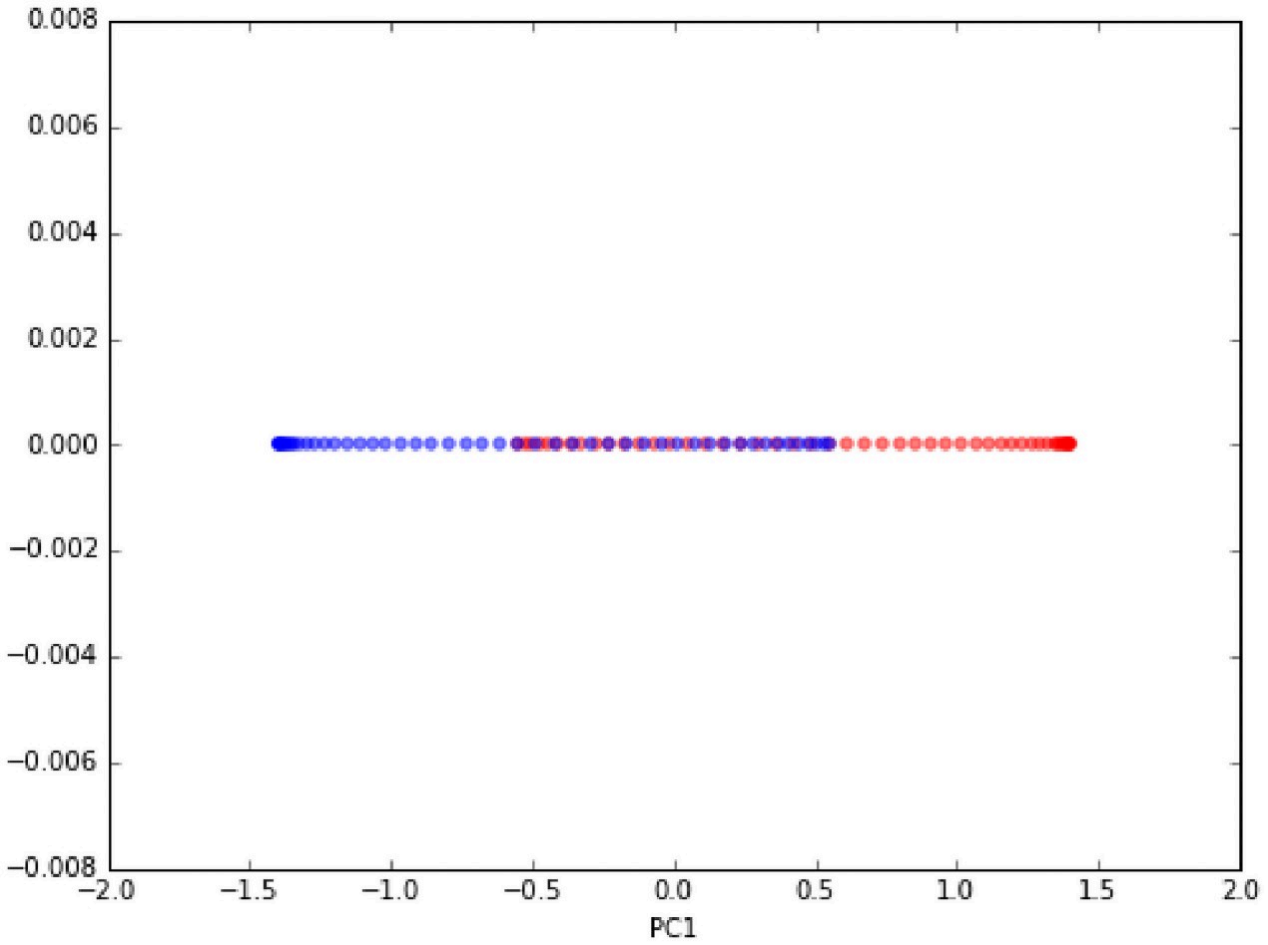
Standard PCA result [69].

**Fig 25:**
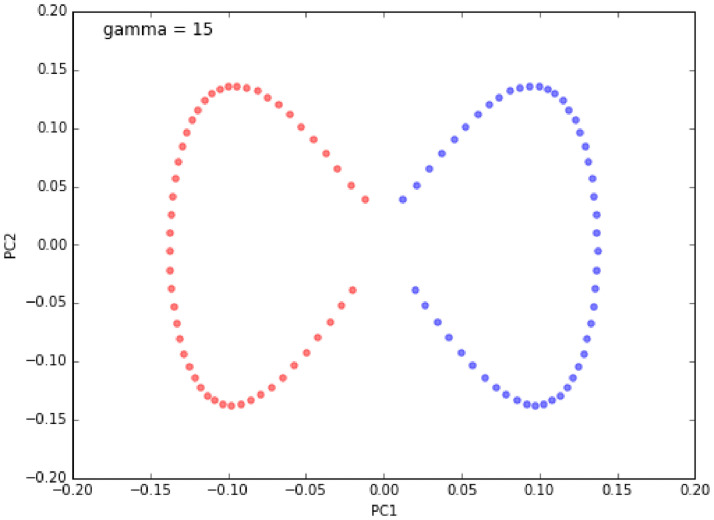
Original data [69].

**Fig 26:**
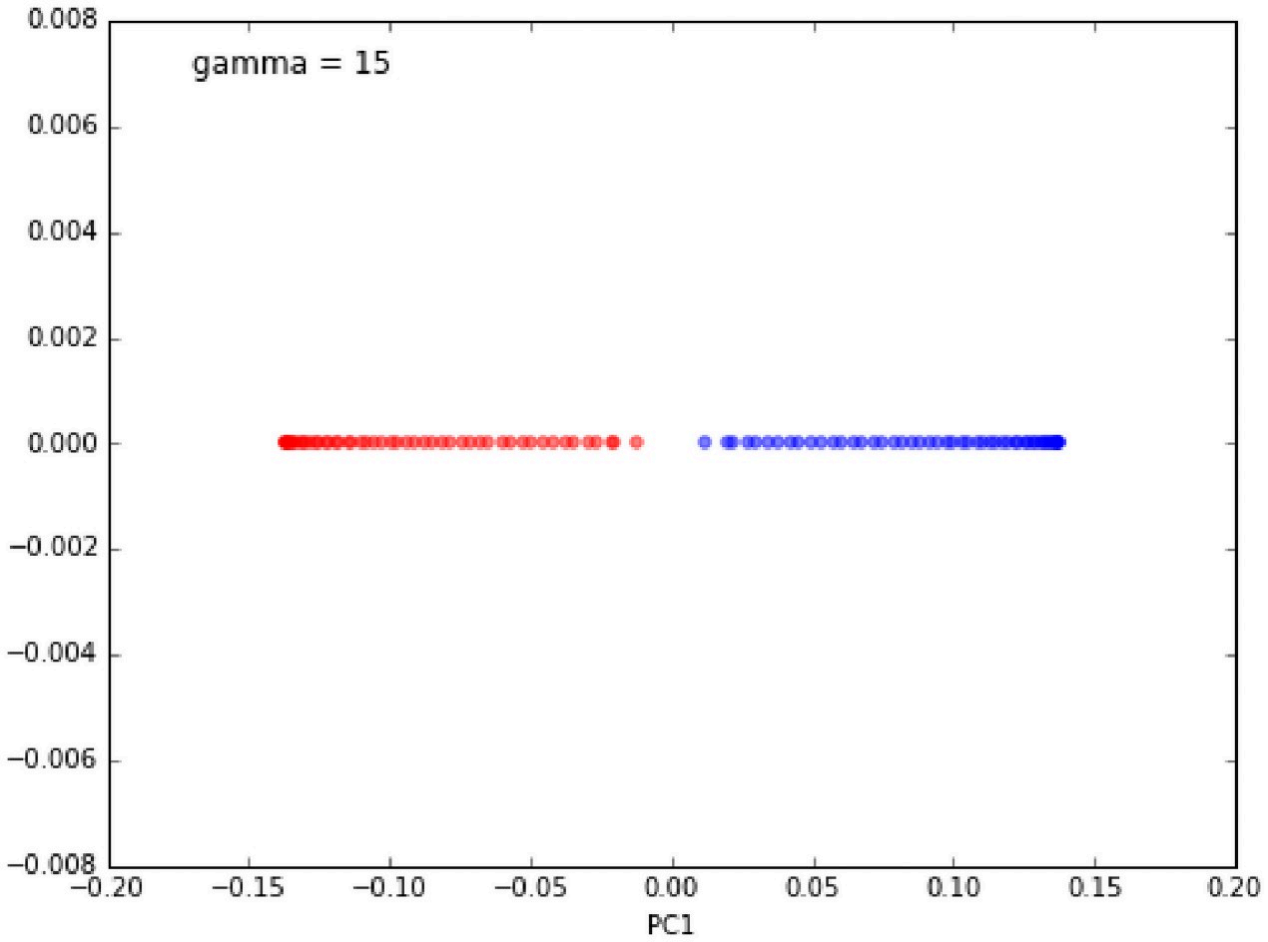
Kernel PCA result [69].

Figs 23–26 show an example of the use of standard PCA and the Gaussian radial basis function (RBF) kernel PCA on a nonlinear dataset. The distribution of data is show in Figs 23 and 25. Figs 24 and 26 are the corresponding results. One can clearly see that the projection via RBF kernel PCA produced a subspace in which the classes are well separated.

#### Verification

**Fig 27:**
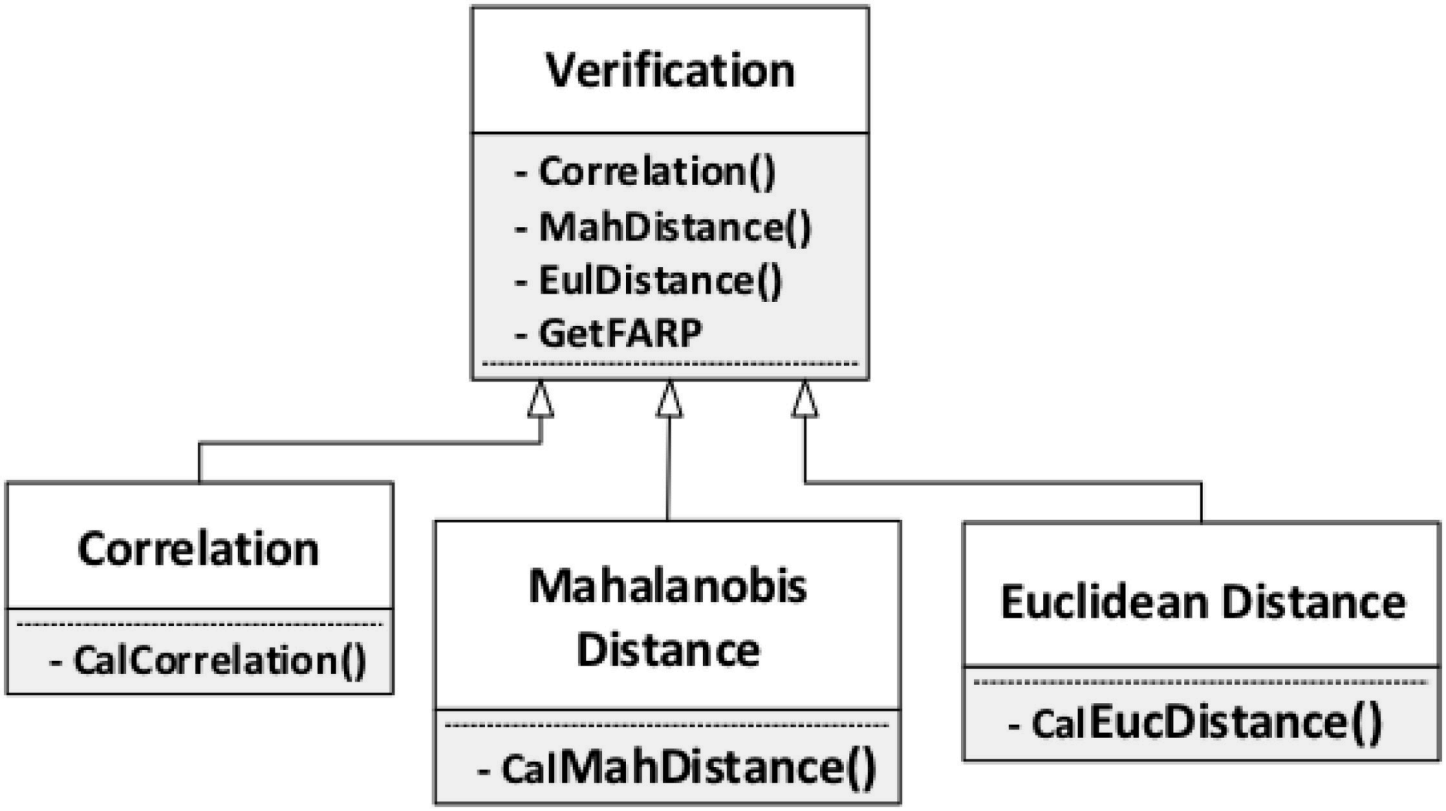
Verification.

Fig 27 shows the three measurements we introduce for the final verification step. The methods in this step are just mathematical formulae related to matrix operations, since the verification step is really to compare the results from previous steps represented as a matrix. The three methods are correlation, Mahalanobis distance, and Euclidean distance.

*Correlation*. A statistical correlation table or graph can describe the relationship and relation direction between two variables, with the exception of the degree of correlation. To solve this, Karl Person, a statistician, proposed a novel correlation coefficient. In general, correlation coefficients are classified into simple, multiple, and classic correlation. Here, we only discuss simple correlation.

The correlation of two variables *X* and *X*′ is calculated as shown in Eq (18).

$$\begin{array}{c} \delta \left(X,X^{\prime }\right)=\frac{E\left(XX^{\prime }\right)-E\left(X\right)E\left(X^{\prime }\right)}{\sigma \left(X\right)\sigma \left(X^{\prime }\right)} \end{array}$$ (18)

Where *E*(*X*) refers to the expectation of the variable *X* and *σ*(*X*) refers to the variance of *X*.

The calculation of a correlation coefficient has low computational complexity, but the result varies depending on the number of samples. For a low number of samples, the result fluctuates significantly with the addition of a new sample; the same is not true for a high number of samples.

*Mahalanobis distance*. P. C. Mahalanobis is creator of the Mahalanobis distance, which expresses the covariance distance of data. Compared with other related measurements, Mahalanobis distance takes the relationship between various features into account. One advantage of this distance over the others is its scale-invariant property, which means it can remove the interference between variables. Eq (19) shows how to calculate the Mahalanobis distance of two vectors.

$$\begin{array}{c} D\left(X_{i},X_{j}\right)=\sqrt{{\left(X_{i}-X_{j}\right)}^{T}S^{-1}\left(X_{i}-X_{j}\right)} \end{array}$$ (19)

Where *S* represents the covariance matrix.

The most significant shortcoming of Mahalanobis distance is that it might exaggerate the impact of variable with small variation.

*Euclidean distance*. The Euclidean distance is a very popular distance metric in statistics. It represents the distance of two vectors in space. In a two dimensional space, the Euclidean distance is calculated as shown in Eq (20).

$$\begin{array}{c} O\left(\rho \right)=\sqrt{{\left(x_{1}-x_{2}\right)}^{2}+{\left(y_{1}-y_{2}\right)}^{2}} \end{array}$$ (20)

When extended to an n-dimensional space, the Euclidean distance of a vector *a*(*X*11, *X*12, …, *X*1*n*) and another vector *b*(*X*21, *X*22, …, *X*2*n*) is calculated as shown in Eq (21).

$$\begin{array}{c} d_{ab}=\sqrt{\sum_{k=1}^{n}{\left(x_{1k}-x_{2k}\right)}^{2}} \end{array}$$ (21)

The Euclidean distance is the simplest measurement of two random variables. However, it does not take into consideration the distribution or relationship among different variables in its calculations. As a result, the euclidean distance may not reflect as much information as other approaches do.

## Case studies

In order to demonstrate the utility of the model proposed in the previous section, this section presents four case studies which utilize different variations of the model. The case studies do not simply select variations from the model. Instead, they show why the variations are chosen and what type of problems can be solved. Furthermore, to achieve optimal performance, for some of the steps in the case studies, the combinations and mutations of the variations are presented.

The first case study targets the construction of a PCA-based face recognition system for smart phones. For this application, the main problems are illumination and facial expression. Therefore, the variations chosen for the model aim at reducing the influences caused by these two factors. The system description and requirements are provided.

The other three case studies focus on the choice of variations. To demonstrate how the model can help users customize their applications, these three case studies select different variations for each step. Furthermore, the reason of choosing the variation, i.e., the type of problems which is solved by the variation, is also provided to help users understand and use the model better. For all of these three case studies, the overview of the system and the demonstration of selecting each variation is presented.

At the end of this section, the variations which are not selected in any of the case studies are discussed. Additionally, the combination of some variations and omission of some steps in the model are also suggested. It should be noticed that the case studies in this section intend to provide a guide to how the model can be used. Nevertheless, since the total number of variations which can be produced by the model exceeds 150, it is not feasible to develop statistical analyses for all cases. To mitigate this, we decided to present the evaluation of this framework in the form of four case studies described in the next sections.

### PCA-based face recognition system for smart phones

#### Description

Face recognition is showing its value on smartphone security, where it finds a more suitable environment for implementation than on desktop computers. Furthermore, the development of the frontal camera for smartphones facilitates the face recognition system, since the photos are always shot with high resolution and from frontal angle. However, there are several salient factors affecting the quality of input images for the face recognition, such as non-uniform illumination, and exaggerated facial expressions, because of the usage habits of smartphones. Therefore, the system of this case study specifically aims at solving the aforementioned two problems.

#### System overview

Targeting to solve the illumination and facial expression problems, the case study selects variations from the model presented in our main section (“Framework for PCA-based Face Recognition”) to build a face recognition system for smart phones. To achieve the best performance, the system does not strictly follow the flow of the model, i.e. for some phases, all variations are used collaboratively; for other phases, none are used.

**Fig 28:**
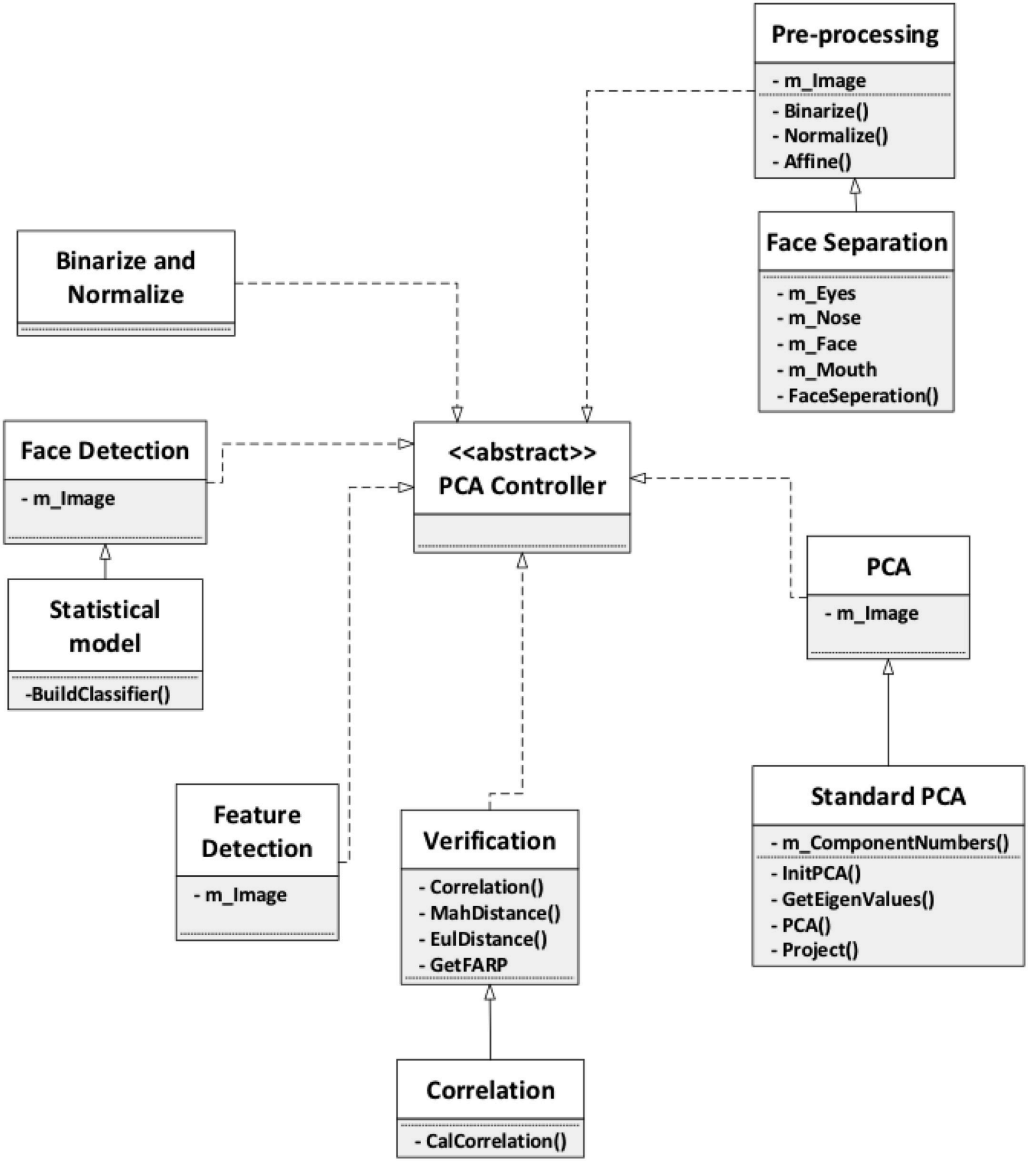
Face recognition for smart phones.

Before applying the model, binarization and normalization are employed to convert the color image to a grey-scale image. The first phase in the model, image representation is omitted in this case study because of the limited computational ability of smartphones. For face detection, a statistical-based approach is selected followed by a feature dectection step. In the pre-processing step, two variations, face separation and LBP, are combined as both illumination and facial expression problems need to be considered. For the PCA step, standard PCA is chosen. Finally, correlation is selected as the verification method. The overview of this face recognition system is presented in Fig 28.

#### Image representation

As the computational capabilities of smartphones are still much lower than desktop PCs, the three face representation methods requiring relatively high computational resources do not work for smartphones. Instead, two simple image processing approaches, which are binarization and normalization are used. They are not able to achieve the effect of the methods in our model in terms of noise reduction or image enhancement, but they do contribute to highlight the significant information of face region. The fast running speed of these two algorithms is the most attractive advantage when applied to smart phones. The codes of binarization and normalization are provided in the S1 Appendix (sections “Image Representation—Binarization” and “Image Representation—Normalization”).

#### Face detection

The face detection used in the system is based on a statistical model whose classifier is based on Haar-like features. The code is shown in the S1 Appendix (section “Face Detection”).

#### Pre-processing

In the context of smart phones, both facial expressions and illumination need to be considered. Therefore, we decide to combine face separation aiming to solve expression problems with LBP which targets illumination problems to achieve better results. The code is in the S1 Appendix (sections “Pre-processing—Face Separation” and “Pre-processing—LBP”).

#### PCA

To achieve optimal performance, kernel PCA always requires a relatively long training time and large training dataset. Nevertheless, smart phone users expect the phone to respond in real-time and the storage capacity of smart phones is also limited. Therefore, we select the simpler standard PCA for the system. The code is shown in the S1 Appendix (section “PCA”).

#### Verification

Among the three verification methods proposed in the model, Mahalanobis distance reflects most similiarity between two images, whereas Euclidean distance uses the least computing resources. For a smart phone environment, we choose correlation as it can be regarded as the compromise which considers not only accuracy but also computational complexity. The code is shown in the S1 Appendix (section “Verification—Correlation”).

### Case Study 2

#### Description

**Fig 29:**
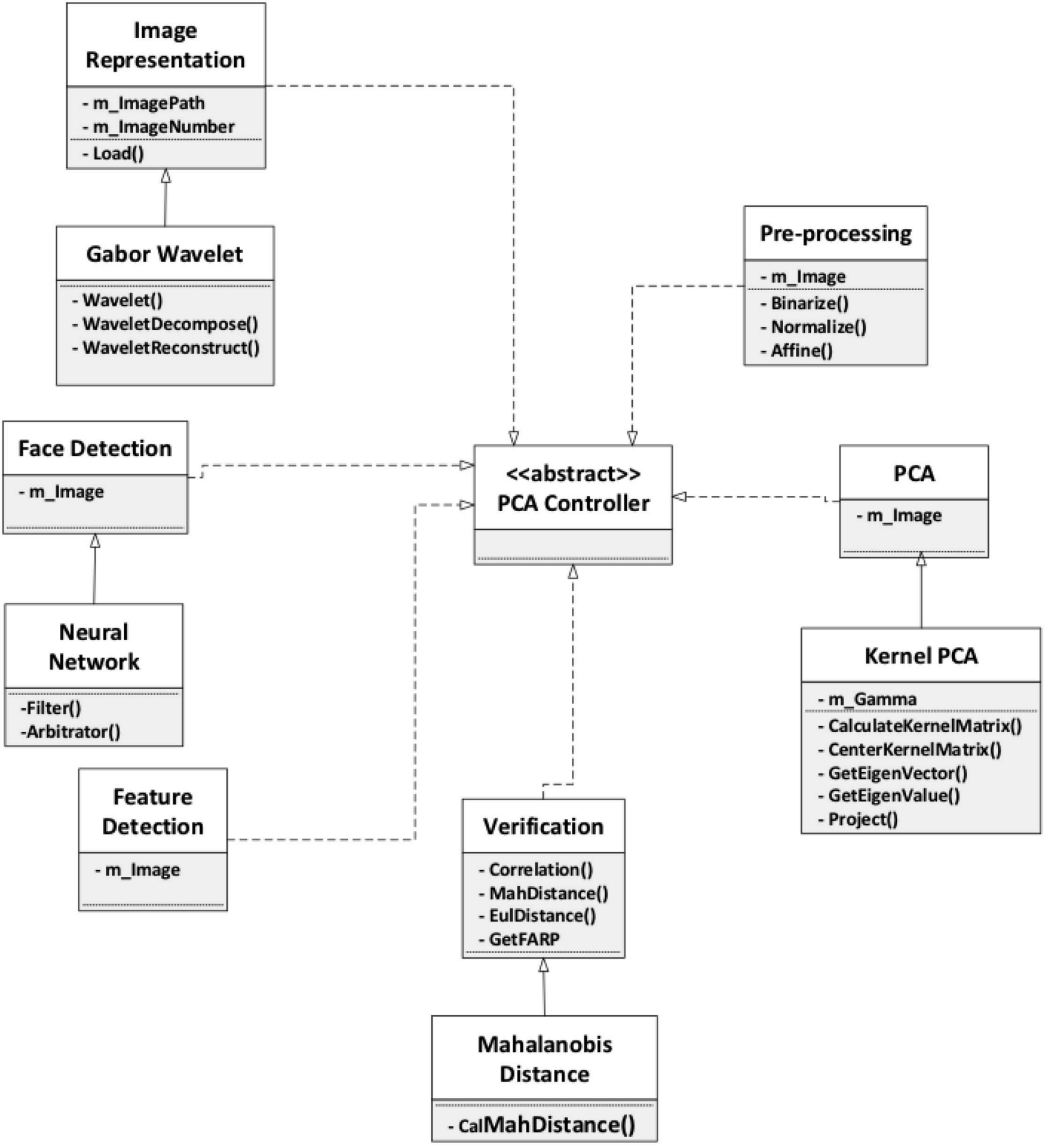
Case Study 2.

In this case study, we intend to select the variations which are not used in the first case study to offer a comprehensive introduction to the model. For face representation, Gabor Wavelet is chosen to extract more precise facial features. To detect face region, a neural network method is used, as its detection accuracy outperforms the other two in the model, if we temporally ignore the computation speed. Similar to the first case study, feature detection is also required for aligning the image via affine transformation, since the alignment of image is important to most PCA-based approaches. Then we skip pre-processing steps, as compared with the standard PCA, kernel PCA is capable of dealing with more complex data (non-linear), so pre-processing might be redundant in this case. At last, Mahalanobis distance is used for verification. The overview of the process is shown in Fig 29. The implementation of the variations selected for this case study is written in C++ and is provided in the S1 Appendix.

#### Face representation

For face recognition systems running on PCs with high-end configuration, the speed of executing algorithms can be ignored, to some extent. Therefore, approaches producing more precise result while costing more computational resources can be used. Therefore, Gabor Wavelet is selected for face representation in this case study, as it is the most complex method, compared to the others in the model, but extracts most useful facial features.

Gabor Wavelet transfers image data from the spatial domain to frequency domain, so it is capable of dealing with noise such as illumination, shooting angle, or occlusion. Moreover, when there are multiple faces appearing in the same image, Gabor Wavelet is sensitive to the distinct features on different faces, which facilitates the later recognition process.

The code to implementing Gabor Wavelet is shown in the S1 Appendix (section “Face Representation—Gabor Wavelet”) and is also available online at: https://blog.csdn.net/carson2005/article/details/40581463.

#### Face detection

The neural network-based face detection method actually belongs to a statistics model-based methods, since it also trains the network by inputing images, which means the more images it tests, the more accurate it becomes. However, as neural network origins from biological knowledge, and its principle and detecting process differs significantly from traditional statistics model-based detection methods, it is always classified in an independent category in face recognition.

Similar to Gabor Wavelet for face representation, neural network-based face detection also costs high computation resources. Therefore, it is suitable for high-end platforms or systems which do not require real-time recognition but has to guarantee high accuracy, such as face recognition system used by the military. Moreover, the neural network-based method is extendable, since the accuracy level can be adjusted by changing the number of layers in the network.

The code in S1 Appendix section entitled “Face Detection” shows a simple BP neural network implementation. The code is available online at: https://blog.csdn.net/xiaowei_cqu/article/details/9027617.

#### PCA

Standard PCA has been demonstrated to be an efficient tool for face recognition, which produces high recognition accuracy and executes quickly. For systems requiring quick response, standard PCA is a good choice. However, there are still some factors which are ignored by standard PCA, such as the non-linear information contained in image data.

Kernel PCA is an extension of PCA using a kernel technique which takes the non-linear information into account. In fact, the non-linear information plays an important role in image data, such as the influence of wearing glasses or having eyes closed or opened. In most face dataset for research experiments, the face images are still taken with limitations. Nevertheless, in practical applications, such as criminal recognition or scene surveillance, the shooting environment might be much worse. In this case, the non-linear component in the image data increases exponentially.

Therefore, in this case study, we select kernel PCA. The code to implement kernel PCA is in the S1 Appendix section “PCA—Kernel PCA”.

#### Verification

Among the three variations in the verification step in the model, Mahalanobis distance is the only measurement that uses a covariance matrix between two data vectors. Therefore, it is more complicated to calculate, but reflects the relationship between different dimensions of the data, which is important when comparing images. In face recognition, applying Mahalanobis distance to the final verification step helps the system choose a more explicit threshold.

The code for calculating Mahalanobis distance is specified in the S1 Appendix section “Verification—Mahalanobis Distance”.

### Other case studies

In this section, two more case studies are presented. The case studies also show the entire workflow and the variations selected from the model as well as the situations in which the variations work well. The detailed implementation, including the C++ code, of the variations is provided in our repository: https://git.uwaterloo.ca/palencar/a_software_framework_for_pca-based_face_recognition.git. Still, aiming to show a comprehensive application of the model, these two case studies present the variations which are not used in previous case studies.

#### Case Study 3

**Fig 30:**
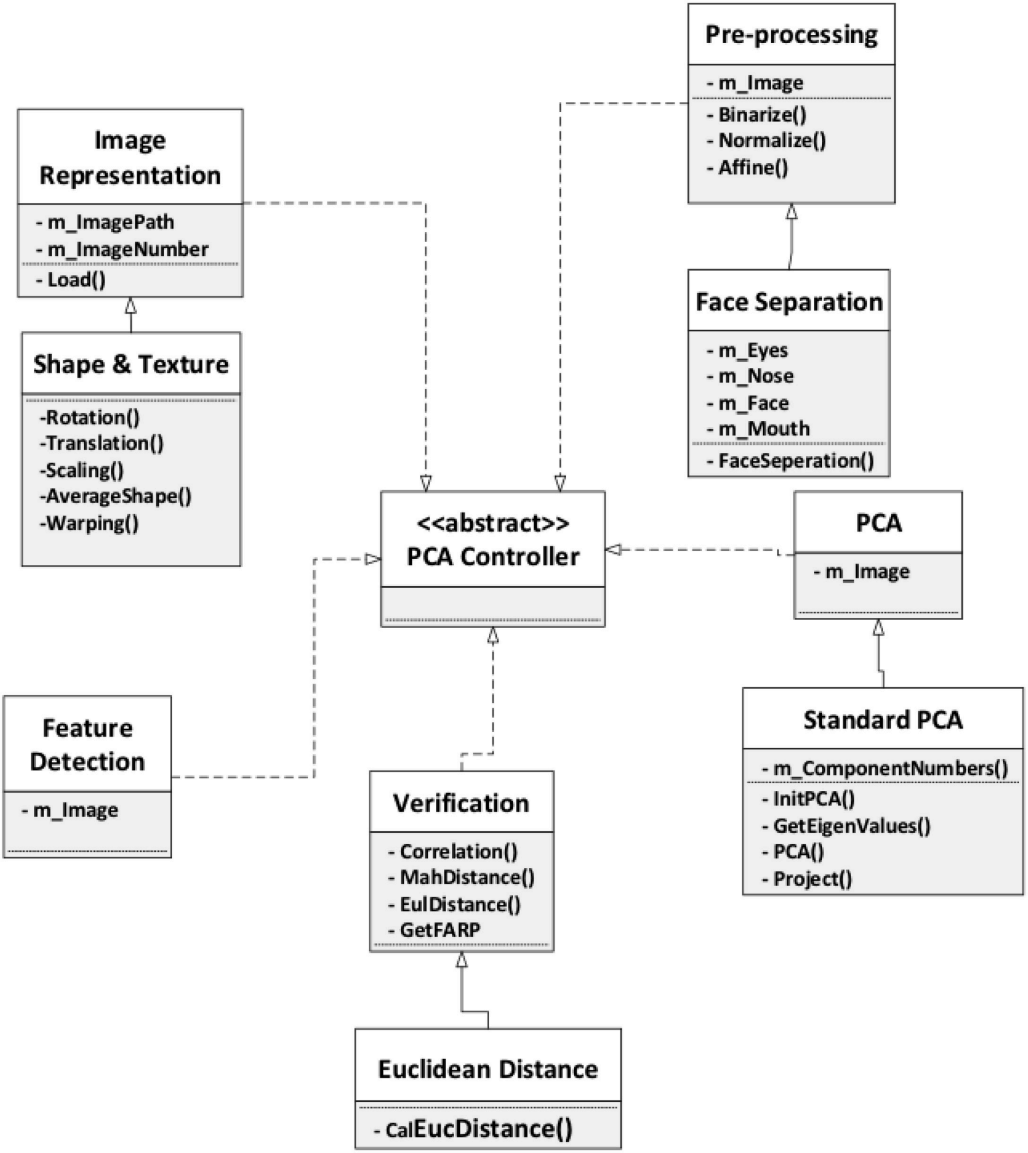
Case Study 3.

*Overview*. In this case study, shape and texture approach is used for image representation and face detection. The feature detection step is required for the following face separation process. Standard PCA is employed as the core recognition approach. Finally, we use Euclidean distance for final verification. Compared with the system built in Case Study 1, this process uses relatively more time and computational resources mainly because of the complexity of the shape and texture approach for face representation. Nevertheless, since the shape and texture approach defines the face region and depicts the face contour precisely, there is no need to detect the face region again, which saves some time. When compared with Case Study 2, this process does not spend time on training the neural network or performing kernel PCA. Though it is not able to achieve the accuracy of Case Study 2, it can be employed for platforms where real-time response is needed. Fig 30 shows the process overview.

*Description*. The first step of the shape and texture approach is to obtain the geometry of the face, which is the shape. The shape is described by a set of manually annotated control points, so the noise of background image is removed in advance. This manual annotation process might be time-consuming; however, once the template is built, the remaining work to be done is just to align other images to the template through series of automatic transformations. Then texture, shape-free image can be generated using a warping transformation. After performing these two steps, a precise face region is catptured; and what is more important is that the face is described precisely without any noise like color or illumination. This process might comsume more time than the other variations do, but it actually combines image representation and face detection, which makes it reasonable.

The pre-processing step and PCA performing step is the same as what happens in Case Study 1, so the details are not presented again. However, because of the precision of face representation provided by shape and texture, PCA is able to generate more precise result as well, though more time is needed as the features extracted by the shape and texture method are more complex.

The Euclidean distance is selected for the final verification step. It is the simplest measurement among all variations in the model and presents the most straightforward relationship between two images. Honestly, it does not provide as much information as the other methods; however, Euclidean distance always collaborates with mathematical operations, such as cosine. After combining together, Euclidean distance is able to increase fluctuation range, if needed, so that the threshold is easily selected.

#### Case Study 4

*Overview*. In this case study, we choose PCA as the image representation approach because of its ability to reduce data dimensions. Then a color-based method is used for detecting face region. We skip the feature detection and pre-processing steps; however, normalization and an affine transformation are required. Kernel PCA is employed as the recognition approach. Finally, Mahalanobis distance is used as verification method.

**Fig 31:**
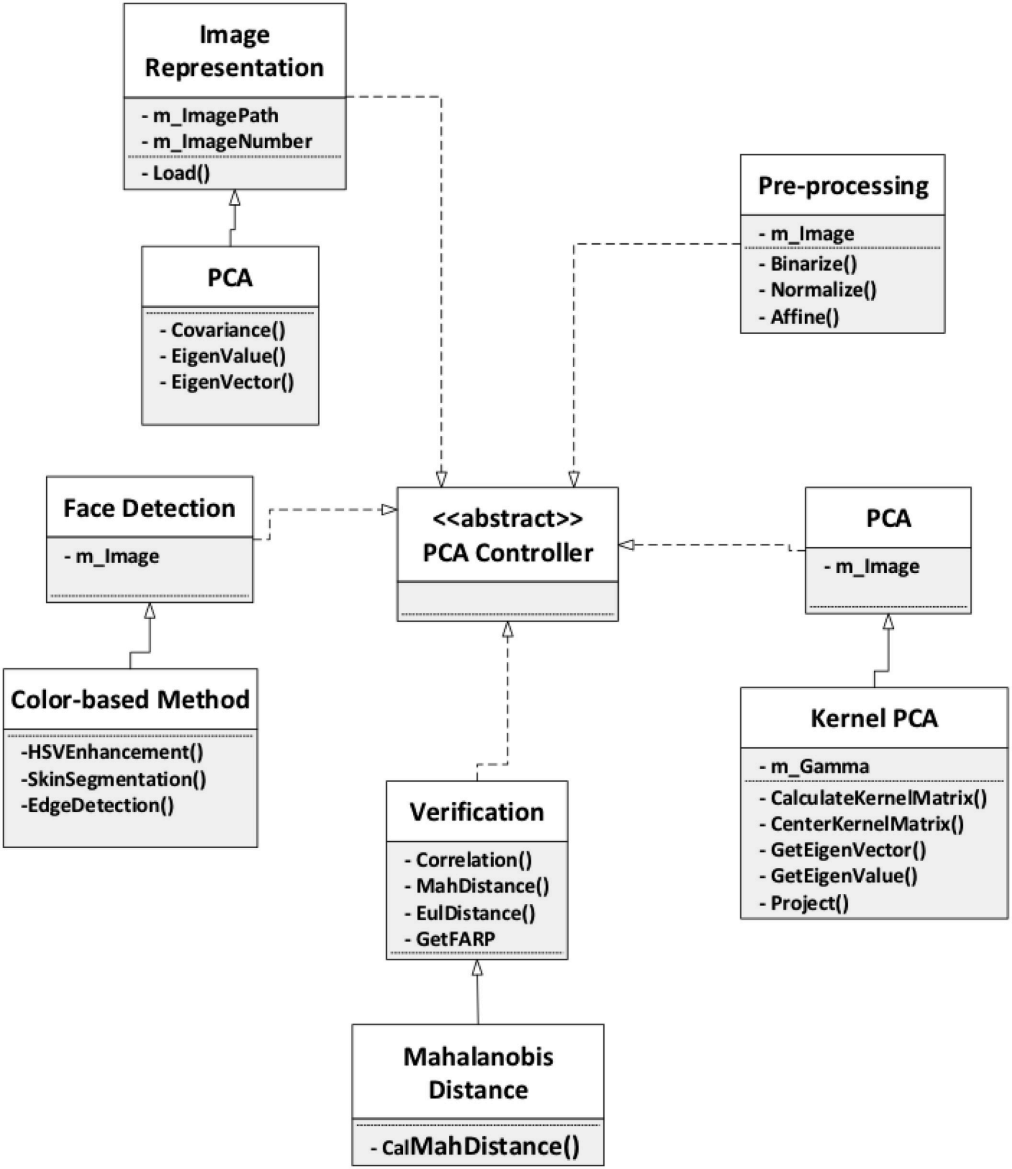
Case Study 4.

Although PCA is performed at the beginning of the process, which might cost more time than other variations, it saves time for the following steps, since PCA reduces the dimensionality of the original image. Color-based method detects face regions based on skin color distribution. The time consumption of this class of methods varies siginificantly depending on how the classifier is built. Therefore, it is flexible for different situations. Overall, this process relies highly on the training process, as most of the steps need training, and the more images are provided, the more robust the system. Therefore, it is suitable for relatively fixed platforms, i.e., the database is set up in the back-end. The response time is short once the system is built and the training is done. The process overview is shown in Fig 31.

*Description*. Although PCA is the core step in our model for the recognition process, it can also be used as an image representation approach. After applying PCA to original images, the data size is substantially reduced while the important information is retained, i.e. the images are compressed without destruction. It undoubtedly facilitates the following process, as the original images are always too large. Furthermore, unlike the PCA employed during the recognition process, compressing images with PCA does not need to project the original images back to the sub-space, so its running time becomes reasonable.

A color-based method is initially inspired by the difference between skin color distribution and background color distribution. It defines a series of prior knowledge, such as black circle region implies pupil candidates, to detect the entire face region. As the statistical model rises in face recognition, researchers integrate color-based methods with a statistical model. Basically, they train the face color model, and compare the new image with the model to generate a detection result. Therefore, the running time of color-based methods varies depending on the complexity of the algorithm. Based on different requirements, color-based methods can be modified.

Normalization and an affine transformation are needed for image alignment. Kernel PCA is selected in this case study, since there is still some non-linear information which is not processed by image representation using PCA. Furthermore, as each step in this process relies on training, the kernel PCA training can be conducted in parallel which does not consume extra time.

### Conclusion

In this section, four case studies are presented to describe how the model works and help users customize their own application. The first case study is for smart phones. The second case study aims to generate the most precise result with the variations of the model. The other two case studies, to some extent, compromise the strengthnesses and weaknesses of the first and second case studies. All variations for each step in the model are covered within these four case studies.

However, because of the length limitation of the paper, it is impossible to present all variations which can be generated from the model. Actually, in practical applications, some steps are omitted, some variations are combined together, and some variations collaborate with other simple mathematical operations. Therefore, the model is able to help users generate a large number of applications based on their requirements.

## Conclusions and future work

### Conclusion

PCA-based face recognition has been studied for decades. Some image processing toolkits like OpenCV have implemeted PCA algorithm and even its associated image processing approaches, which provide significant help for software developers in this field. However, setting up a PCA-based face recognition system is still time consuming, especially when adapting to different types of image data or fitting various situations, such as non-uniform illumination, exaggerated facial expression, or shooting angle. The existing tools can hardly help users quickly customize their own applications, since the requirement of different systems are quite variable. Searching for the implementation of an algorithm from the toolkit and integrating it with the current application can produce a lot of pain for developers. Therefore, a tool which can help developers establish their systems and select optimal approaches for each step in the process is critical.

The framework describes the entire workflow of the system, and provides multiple variations for each step to fit different situations and help software developers customize their own applications. With the framework, developers are allowed to establish their system at a higher level, i.e the straightforward implemention details are handled by the framework.

The main conclusions of this study are the large number of variations of the framework (150) and its assistance to software developers, non-expert researchers, and domain experts in the field of face recognition. These conclusions are drawn from the data as explained in the following paragraphs.

The framework provides more than 150 supported variations in total for different situations and satisfying various requirements. This number is derived from the multiple options for each of the six steps of the PCA-based face recognition process. There are at least two variations provided for each step in the process, so that developers can select the optimal one for their own purpose. Moreover, some of the variations can be combined to achieve better performance. The architecture of the framework is also flexible, which means some of the steps can be omitted when being applied to specific cases. Certainly, because of its flexibility, attaching more variations to the model is possible.

The framework offers a significant help to software developers, non-expert researchers, and domain experts in the field of face recognition, since the 150 supported variations produced by the framework cover a number of requirements for face recognition applications. These roles are highlighted based on the initial and advanced experience levels they have with the field of face recognition and the utility of the framework for them, as explained in the case studies in the previous section.

Inexperienced developers who are not familiar with face recognition can use the framework as a guide when they build applications, since the entire PCA-based face recognition process is described explicitly. They can learn from the framework and then modify or extend the basic process to meet their specific design requirements.

For non-expert researchers who are familiar with the process of PCA-based face recognition but do not have too much knowledge on specific techniques for each step, the variations in the framework helps significantly. The properties of most variations are demonstrated in the Case Study Section, which can be used as a guide to assist the non-expert researchers to select the optimal approach for particular requirements.

For domain experts who are experienced in face recognition, designing the structure of an application or selecting the best approach for each step is not the major problem. However, implementing the application is time-consuming. In this case, the framework provides the complete implementation for each variation, which saves time for domain experts.

As an example, when mobile phone application developers build a face recognition application for smart phones for the first time, i.e., they are inexperienced in this field, the major problem is the lack of domain knowledge. In this case, the framework is able to give them a straightforward guide about PCA-based face recognition which can inspire them so that they can easily start implementing. The variations will also assist them throughout the implementing process.

Nevertheless, for non-expert researchers who want to build a face recognition application used for security, the major problem becomes selecting the best approaches to achieve the optimal recognition accuracy. In this case, with the demonstration of each variation, the reasearchers can find a variation of the framework that best matches their application. Morover, for both examples, the implementation details are handled by the framework, which significantly improves efficiency.

The paper presents four case studies which cover some of the variations. The case studies intend to offer a straightforward impression on how the framework can help developers establish their applications. However, the framework is capable of dealing with many more complicated situations than shown in the case studies.

### Future work

The framework proposed in the paper provides a prototype or starting point of our thinking. There is some potential future work involving the implementation of the framework, the enhancement of the framework, and practical case studies, which can be considered.

First, the implementation of the framework could provide a friendly user interface in which all variations proposed in the framework are modularized so that, developers can build their systems by simply dragging the variations and connecting them with lines. Furthermore, the interface will not only reduce implementation time for developers but also help them select the optimal approaches to achieve best result.

Second, with the development of face recognition field, more advanced techniques are proposed, which might outperform the algorithms that we have already included in the framework. Therefore, studying new techniques and integrating them with our framework would be meaningful and help us improve the comprehensiveness of the framework. Certainly, it is not enough to just add new variations to the model. Classifying those variations by their distinct functions is more important, as users would then be able to choose the variations that they need for fitting their own applications. Similarly, the framework can be extended to support other types of techniques as they become available to the field of face recognition, such as neural networks.

Third, this framework is based on PCA because of its popularity and the problems that still arise from its use in face recognition. However, other feature extraction algorithms such as SURF [70] or SIFT [71] or their variations can be used. A study on the impacts of the use of other techniques for feature extraction and the adaptations that are necessary to be used in our framework is valuable, will augment the face recognition process, and enable new face recognition applications.

Fourth, the framework can be provided an architectural design based on a layered architecture. The architecture may contain three layers, which can include (i) data acquisition, (ii) data processing, and (iii) face image classification and decision-making. An architectural design would emphasize the data flow and show more about how the framework works in terms of its components.

Fifth, the framework can be further evaluated in terms of a comparative performance analysis. For example, a comparative performance analysis could be performed assess the framework performance in the case different PCA based techniques are used (e.g. standard and kernel PCA).

Last, conducting case studies in the practical context can help us verify the efficiency and usefulness of the framework and detect potential defects.

## Supporting information

S1 Appendix (PDF)
