# Supplementary material for: A face recognition software framework based on principal component analysis
Source: PLoS One. 2021 Jul 22;16(7):e0254965. doi: 10.1371/journal.pone.0254965 (PMC8384131; doi:10.1371/journal.pone.0254965)
Supplement: S1 Appendix — (PDF) [file pone.0254965.s001.pdf]

Image Representation - Binarization

The functions cvLoadImage() and cvtColor() are integrated in OpenCV.

```
tmpImg = cvLoadImage(c);
tmpImgCopy = cvLoadImage(c);

Mat matTmpImgCopy(tmpImgCopy,0);
Mat frame_gray(matTmpImgCopy.rows, matTmpImgCopy.cols, matTmpImgCopy.depth
    ());
cvtColor( matTmpImgCopy, frame_gray, CV_BGR2GRAY );
tmpImgCopy2 = frame_gray;
```

Image Representation - Normalization

The function normalize() is integrated in OpenCV.

```
Mat CPPPCA::normalize(const Mat& src) {
    Mat srcnorm;
    cv::normalize(src, srcnorm, 0, 255, NORM_MINMAX, CV_8UC1);
    return srcnorm;

}    // end of void CPPPCA::normalize()
```

Face Detection

The parameter strClassifierFileName reads in the file path of the classifier file. Then function detect_and_draw() configures the classifier.

```
BOOL CPPPCA::FaceProc(IplImage* imgSource)
{
    const char* strClassifierFileName = "";
    static CvHaarClassifierCascade* cascade = 0;

    cascade = (CvHaarClassifierCascade*)cvLoad(strClassifierFileName);

    CvMemStorage* storage = cvCreateMemStorage(0);
    detect_and_draw(imgSource, storage, cascade);

    if(m_faces->total != 0){
        m_FaceRect.x = (*(CvRect*)cvGetSeqElem( m_faces, 0)).x;
        m_FaceRect.y = (*(CvRect*)cvGetSeqElem( m_faces, 0)).y;
        m_FaceRect.width = (*(CvRect*)cvGetSeqElem( m_faces, 0)).width;
        m_FaceRect.height = (*(CvRect*)cvGetSeqElem( m_faces, 0)).height;

        cvClearMemStorage( storage );

        return TRUE;
    }
    else
    return FALSE;

}    // end of void CPPPCA::FaceProc()

void CPPPCA::detect_and_draw( IplImage* img, CvMemStorage* storage,
    CvHaarClassifierCascade* cascade )
{
    static CvScalar colors[] =
    {
        {{0,0,255}},
        {{0,128,255}},
```

```
        {{0,255,255}},
        {{0,255,0}},
        {{255,128,0}},
        {{255,255,0}},
        {{255,0,0}},
        {{255,0,255}}
    };

    double scale = 1;
    IplImage* gray = cvCreateImage( cvSize(img->width,img->height), 8, 1 );
    IplImage* small_img = cvCreateImage( cvSize( cvRound (img->width/scale),
    cvRound (img->height/scale)),
    8, 1 );
    int i;

    cvResize( img, small_img, CV_INTER_LINEAR );
    cvClearMemStorage( storage );

    if( cascade )
    {
        double t = (double)cvGetTickCount();
        m_faces = cvHaarDetectObjects( small_img, cascade, storage,
        1.1, 2, 0
        cvSize(30, 30) );

        t = (double)cvGetTickCount() - t;
        printf( "detection time = %gms\n", t/((double)cvGetTickFrequency()*1000.) );
        for( i = 0; i < (m_faces ? m_faces->total : 0); i++ )
        {
            CvRect* r = (CvRect*)cvGetSeqElem( m_faces, i );
            CvPoint center;
            int radius;
            center.x = cvRound((r->x + r->width*0.5)*scale);
            center.y = cvRound((r->y + r->height*0.5)*scale);
            radius = cvRound((r->width + r->height)*0.25*scale);
        }
    }

    cvReleaseImage( &gray );
    cvReleaseImage( &small_img );

}    // end of void CPPPCA::detect_and_draw()
```

Pre-processing - Face Separation

The four Rect type parameters roi, eyesRoi, mouthRoi, NoseRoi define the face area, eyes area, mouth area, and nose area respectively.

```
Mat warp_dst = GetAffinedMat(tmpImgCopy2, frame_gray);

IplImage *imgLbpSrc = (&(IplImage)warp_dst);
IplImage *imgLbpDst = cvCreateImage(cvGetSize(imgLbpSrc), IPL_DEPTH_8U,1);;

m_lbpInst.CreatLBP(imgLbpSrc, imgLbpDst);

Mat lbp_dst(imgLbpDst);

Rect roi(51, 19, 80, 89);
Mat matRoi = lbp_dst(roi);
m_vmatLbpFace.push_back(matRoi);
```

```

Rect eyesRoi(46, 22, 84, 38);
Mat matEyes = lbp_dst(eyesRoi);
m_vmatLbpEyes.push_back(matEyes);

Rect mouthRoi(69 ,102, 38, 22);
Mat matMouth = lbp_dst(mouthRoi);
m_vmatLbpMouth.push_back(matMouth);

Rect NoseRoi(71, 57, 39, 37);
Mat matNose = lbp_dst(NoseRoi);
m_vmatLbpNose.push_back(matNose);
```

Pre-processing - LBP

```
void CLBP::CreatLBP(IplImage *src,IplImage *dst)
{
    int iTemp[8] = {0};
    CvScalar s;

    IplImage *ImgTemp = cvCreateImage(cvGetSize(src), IPL_DEPTH_8U, 1);
    uchar *data = (uchar*)src->imageData;
    int iStep = src-> widthStep;

    for (int i=1;i<src->height-1;i++)
    for(int j=1;j<src->width-1;j++){

        int sum=0;
        if(data[(i-1)*iStep+j-1]>data[i*iStep+j])
        iTemp[0]=1;
        else
        iTemp[0]=0;
        if(data[i*iStep+(j-1)]>data[i*iStep+j])
        iTemp[1]=1;
        else
        iTemp[1]=0;
        if(data[(i+1)*iStep+(j-1)]>data[i*iStep+j])
        iTemp[2]=1;
        else
        iTemp[2]=0;
        if (data[(i+1)*iStep+j]>data[i*iStep+j])
        iTemp[3]=1;
        else
        iTemp[3]=0;
        if (data[(i+1)*iStep+(j+1)]>data[i*iStep+j])
        iTemp[4]=1;
        else
        iTemp[4]=0;
        if(data[i*iStep+(j+1)]>data[i*iStep+j])
        iTemp[5]=1;
        else
        iTemp[5]=0;
        if(data[(i-1)*iStep+(j+1)]>data[i*iStep+j])
        iTemp[6]=1;
        else
        iTemp[6]=0;
        if(data[(i-1)*iStep+j]>data[i*iStep+j])
        iTemp[7]=1;
        else
        iTemp[7]=0;
        s.val[0] = (iTemp[0]*1+iTemp[1]*2+iTemp[2]*4+iTemp[3]*8+iTemp
            [4]*16+iTemp[5]*32+iTemp[6]*64+iTemp[7]*128);

```

```
        cvSet2D(dst,i,j,s);

    }

}   // end of CLBP::CreatLBP()
```

PCA

The function InitPCA() sets tup the PCA instance for further use. The function GetEigenValues() obtains the eigenvalue of a corresponding eigenvector.

```
void CPPPCA::InitPCA(String strDirName, Mat matSrc, vector<Mat> vmatSrc)
{
    double dbnumber_principal_compent = 0.95;
    Mat pcaImg, projectImg;

    PCA pca(matSrc, Mat(), CV_PCA_DATA_AS_COL, dbnumber_principal_compent);
    Mat EigenVectors = pca.eigenvectors;

    pcaImg = normalize(pca.eigenvectors.row(0)).reshape(1, vmatSrc[0].rows);
    //    pcaFace = pca.eigenvectors.row(0).reshape(1, src[0].rows);
    imwrite((("\\") + strDirName + ("\\Pca.jpg")), pcaImg);

    Mat EigenValues = pca.eigenvalues;

    Mat dst;
    dst = pca.project(matSrc.col(0));
    projectImg = normalize(pca.backProject(dst).col(0)).reshape(1, vmatSrc[0].rows);
    imwrite((("") + strDirName + ("\\Project.jpg")), projectImg);

    m_pcaTrain = pca;

}   // end of CPPPCA::InitPCA()

void CPPPCA::GetEigenValues(String strDirName, Mat matSrc, vector<Mat> vmatSrc, int iImgNum)
{
    string strInt;
    Mat dst, projectImg;
    const string strEigenValue = "D:\\Summer Project\\EigenValue\\FaceEigenValues.xml";
    FileStorage fs(strEigenValue, FileStorage::WRITE);

    for(int i = 0; i < iImgNum; i++){
        strInt = inttostring(i);
        dst = m_pcaTrain.project(matSrc.col(i));

        //        projectImg = normalize(m_pcaTrain.backProject(dst).col(0)).reshape(1, vmatSrc[0].rows);
        //        imwrite((("D:\\Summer Project\\") + strDirName + ("\\Project") + strInt + (".jpg")), projectImg);
        fs << "eigenvalue" + strInt << dst;
    }
    fs.release();

}   // end of CPPPCA::GetEigenValues()
```

Face Representation - Gabor Wavelet

The function WDT() implements Wavelet transformation. The function IWDT() implements inverse Wavelet transformation. The function Wavelet() generates different types of Wavelet. Currently only haar and sym2 Wavelet are implemented. The function WaveletDecompose() implements Wavelet decomposition. The function WaveletReconstruct() implements Wavelet reconstruction.

```
Mat WDT( const Mat &_src, const string _wname, const int _level )const
{
    int reValue = THID_ERR_NONE;
    Mat src = Mat_<float>(_src);
    Mat dst = Mat::zeros( src.rows, src.cols, src.type() );
    int N = src.rows;
    int D = src.cols;

    Mat lowFilter;
    Mat highFilter;
    wavelet( _wname, lowFilter, highFilter );

    int t=1;
    int row = N;
    int col = D;

    while( t<=_level )
    {
        for( int i=0; i<row; i++ )
        {
            Mat oneRow = Mat::zeros( 1,col, src.type() );
            for ( int j=0; j<col; j++ )
            {
                oneRow.at<float>(0,j) = src.at<float>(i,j);
            }
            oneRow = waveletDecompose( oneRow, lowFilter, highFilter );
            for ( int j=0; j<col; j++ )
            {
                dst.at<float>(i,j) = oneRow.at<float>(0,j);
            }
        }

        #if 0
        //normalize( dst, dst, 0, 255, NORM_MINMAX );
        IplImage dstImg1 = IplImage(dst);
        cvSaveImage( "dst.jpg", &dstImg1 );
        #endif
        for ( int j=0; j<col; j++ )
        {
            Mat oneCol = Mat::zeros( row, 1, src.type() );
            for ( int i=0; i<row; i++ )
            {
                oneCol.at<float>(i,0) = dst.at<float>(i,j);
            }
            oneCol = ( waveletDecompose( oneCol.t(), lowFilter, highFilter ) ).t();

            for ( int i=0; i<row; i++ )
            {
                dst.at<float>(i,j) = oneCol.at<float>(i,0);
            }
        }

        #if 0
        //normalize( dst, dst, 0, 255, NORM_MINMAX );
        IplImage dstImg2 = IplImage(dst);
        cvSaveImage( "dst.jpg", &dstImg2 );
```

```
        #endif

        row /= 2;
        col /=2;
        t++;
        src = dst;
    }

    return dst;
}
Mat IWDT( const Mat &_src, const string _wname, const int _level )const
{
    int reValue = THID_ERR_NONE;
    Mat src = Mat_<float>(_src);
    Mat dst = Mat::zeros( src.rows, src.cols, src.type() );
    int N = src.rows;
    int D = src.cols;

    Mat lowFilter;
    Mat highFilter;
    wavelet( _wname, lowFilter, highFilter );

    int t=1;
    int row = N/std::pow( 2., _level-1);
    int col = D/std::pow(2., _level-1);

    while ( row<=N && col<=D )
    {
        for ( int j=0; j<col; j++ )
        {
            Mat oneCol = Mat::zeros( row, 1, src.type() );
            for ( int i=0; i<row; i++ )
            {
                oneCol.at<float>(i,0) = src.at<float>(i,j);
            }
            oneCol = ( waveletReconstruct( oneCol.t(), lowFilter,
                highFilter ) ).t();

            for ( int i=0; i<row; i++ )
            {
                dst.at<float>(i,j) = oneCol.at<float>(i,0);
            }
        }

        #if 0
        //normalize( dst, dst, 0, 255, NORM_MINMAX );
        IplImage dstImg2 = IplImage(dst);
        cvSaveImage( "dst.jpg", &dstImg2 );
        #endif
        for( int i=0; i<row; i++ )
        {
            Mat oneRow = Mat::zeros( 1,col, src.type() );
            for ( int j=0; j<col; j++ )
            {
                oneRow.at<float>(0,j) = dst.at<float>(i,j);
            }
            oneRow = waveletReconstruct( oneRow, lowFilter, highFilter );
            for ( int j=0; j<col; j++ )
            {
                dst.at<float>(i,j) = oneRow.at<float>(0,j);
            }
        }

        #if 0
        //normalize( dst, dst, 0, 255, NORM_MINMAX );
        IplImage dstImg1 = IplImage(dst);
```

```
        cvSaveImage( "dst.jpg", &dstImg1 );
        #endif

        row *= 2;
        col *= 2;
        src = dst;
    }

    return dst;
}
void wavelet( const string _wname, Mat &_lowFilter, Mat &_highFilter ) const
{
    if ( _wname=="haar" || _wname=="db1" )
    {
        int N = 2;
        _lowFilter = Mat::zeros( 1, N, CV_32F );
        _highFilter = Mat::zeros( 1, N, CV_32F );

        _lowFilter.at<float>(0, 0) = 1/sqrtf(N);
        _lowFilter.at<float>(0, 1) = 1/sqrtf(N);

        _highFilter.at<float>(0, 0) = -1/sqrtf(N);
        _highFilter.at<float>(0, 1) = 1/sqrtf(N);
    }
    if ( _wname =="sym2" )
    {
        int N = 4;
        float h[] = {-0.483, 0.836, -0.224, -0.129 };
        float l[] = {-0.129, 0.224,    0.837, 0.483 };

        _lowFilter = Mat::zeros( 1, N, CV_32F );
        _highFilter = Mat::zeros( 1, N, CV_32F );

        for ( int i=0; i<N; i++ )
        {
            _lowFilter.at<float>(0, i) = l[i];
            _highFilter.at<float>(0, i) = h[i];
        }

    }
}
Mat waveletDecompose( const Mat &_src, const Mat &_lowFilter, const Mat &_highFilter )const
{
    assert( _src.rows==1 && _lowFilter.rows==1 && _highFilter.rows==1 );
    assert( _src.cols>=_lowFilter.cols && _src.cols>=_highFilter.cols );
    Mat &src = Mat_<float>(_src);

    int D = src.cols;

    Mat &lowFilter = Mat_<float>(_lowFilter);
    Mat &highFilter = Mat_<float>(_highFilter);

    Mat dst1 = Mat::zeros( 1, D, src.type() );
    Mat dst2 = Mat::zeros( 1, D, src.type()  );

    filter2D( src, dst1, -1, lowFilter );
    filter2D( src, dst2, -1, highFilter );

    Mat downDst1 = Mat::zeros( 1, D/2, src.type() );
    Mat downDst2 = Mat::zeros( 1, D/2, src.type() );

    resize( dst1, downDst1, downDst1.size() );
    resize( dst2, downDst2, downDst2.size() );

```

```
    for ( int i=0; i<D/2; i++ )
    {
        src.at<float>(0, i) = downDst1.at<float>( 0, i );
        src.at<float>(0, i+D/2) = downDst2.at<float>( 0, i );
    }

    return src;
}
Mat waveletReconstruct( const Mat &_src, const Mat &_lowFilter, const Mat
    &_highFilter )const
{
    assert( _src.rows==1 && _lowFilter.rows==1 && _highFilter.rows==1 );
    assert( _src.cols>=_lowFilter.cols && _src.cols>=_highFilter.cols );
    Mat &src = Mat_<float>(_src);

    int D = src.cols;

    Mat &lowFilter = Mat_<float>(_lowFilter);
    Mat &highFilter = Mat_<float>(_highFilter);

    Mat Up1 = Mat::zeros( 1, D, src.type() );
    Mat Up2 = Mat::zeros( 1, D, src.type() );

    Mat roi1( src, Rect(0, 0, D/2, 1) );
    Mat roi2( src, Rect(D/2, 0, D/2, 1) );
    resize( roi1, Up1, Up1.size(), 0, 0, INTER_CUBIC );
    resize( roi2, Up2, Up2.size(), 0, 0, INTER_CUBIC );

    Mat dst1 = Mat::zeros( 1, D, src.type() );
    Mat dst2= Mat::zeros( 1, D, src.type() );
    filter2D( Up1, dst1, -1, lowFilter );
    filter2D( Up2, dst2, -1, highFilter );

    dst1 = dst1 + dst2;

    return dst1;
}
```

Face Detection

The parameter params contains all set up information of the BP network. The parameter layerSizes stands for the number of layers in the network. Here, the network has three hidden layers with an input layer and an output layer. The function predict() enables predicting new nodes in the network.

```
int main()
{
    CvANN_MLP bp;
    CvANN_MLP_TrainParams params;

    params.train_method=CvANN_MLP_TrainParams::BACKPROP;
    params.bp_dw_scale=0.1;
    params.bp_moment_scale=0.1;
    float labels[3][5] = {{0,0,0,0,0},{1,1,1,1,1},{0,0,0,0,0}};
    Mat labelsMat(3, 5, CV_32FC1, labels);

    float trainingData[3][5] = { {1,2,3,4,5},{111,112,113,114,115},
        {21,22,23,24,25} };
    Mat trainingDataMat(3, 5, CV_32FC1, trainingData);
    Mat layerSizes=(Mat_<int>(1,5) << 5,2,2,2,5);
    bp.create(layerSizes,CvANN_MLP::SIGMOID_SYM);//CvANN_MLP::SIGMOID_SYM
    //CvANN_MLP::GAUSSIAN
    //CvANN_MLP::IDENTITY
```

```
    bp.train(trainingDataMat, labelsMat, Mat(),Mat(), params);


    int width = 512, height = 512;
    Mat image = Mat::zeros(height, width, CV_8UC3);
    Vec3b green(0,255,0), blue (255,0,0);

    for (int i = 0; i < image.rows; ++i)
    for (int j = 0; j < image.cols; ++j)
    {
        Mat sampleMat = (Mat_<float >(1,5) << i,j,0,0,0);
        Mat responseMat;
        bp.predict(sampleMat,responseMat);
        float* p=responseMat.ptr<float >(0);
        float response=0.0f;
        for(int k=0;k<5;i++){
            //  cout<<p[k]<<" ";
            response+=p[k];
        }
        if (response >2)
        image.at<Vec3b>(j, i)  = green;
        else
        image.at<Vec3b>(j, i)  = blue;
    }

    // Show the training data
    int thickness = -1;
    int lineType = 8;
    circle( image, Point(501,  10), 5, Scalar(  0,   0,   0), thickness,
        lineType);
    circle( image, Point(255,  10), 5, Scalar(255, 255, 255), thickness,
        lineType);
    circle( image, Point(501, 255), 5, Scalar(255, 255, 255), thickness,
        lineType);
    circle( image, Point( 10, 501), 5, Scalar(255, 255, 255), thickness,
        lineType);

    imwrite("result.png", image);        // save the image

    imshow("BP Simple Example", image); // show it to the user
    waitKey(0);

}
```

PCA - Kernel PCA

The function kernel() works as a switch of two types of mainstream kernels, which are RBF and Polynomial. The function run_pca() implements all main steps of kernel PCA which are calculating the kernel matrix, centering the kernel matrix, and obtaining eigenvectors and eigenvalues.

```
void PCA::load_data(const char* data, char sep){

    unsigned int row = 0;
    ifstream file(data);
    if(file.is_open()){
        string line,token;
        while(getline(file, line)){
            stringstream tmp(line);
            unsigned int col = 0;
            while(getline(tmp, token, sep)){
                if(X.rows() < row+1){
                    X.conservativeResize(row+1,X.cols());
```

```
            }
            if(X.cols() < col+1){
                X.conservativeResize(X.rows(),col+1);
            }
            X(row,col) = atof(token.c_str());
            col++;
        }
        row++;
    }
    file.close();
    Xcentered.resize(X.rows(),X.cols());
}else{
    cout << "Failed to read file " << data << endl;
}

}

double PCA::kernel(const VectorXd& a, const VectorXd& b){


    switch(kernel_type){
        case 2  :
        return(pow(a.dot(b)+constant,order));
        default :
        return(exp(-gamma*((a-b).squaredNorm())));
    }

}

void PCA::run_kpca(){


    K.resize(X.rows(),X.rows());
    for(unsigned int i = 0; i < X.rows(); i++){
        for(unsigned int j = i; j < X.rows(); j++){
            K(i,j) = K(j,i) = kernel(X.row(i),X.row(j));
            //printf("k(%i,%i) = %f\n",i,j,K(i,j));
        }
    }
    EigenSolver<MatrixXd> edecomp(K);
    eigenvalues = edecomp.eigenvalues().real();
    eigenvectors = edecomp.eigenvectors().real();
    cumulative.resize(eigenvalues.rows());
    vector<pair<double,VectorXd> > eigen_pairs;
    double c = 0.0;
    for(unsigned int i = 0; i < eigenvectors.cols(); i++){
        if(normalise){
            double norm = eigenvectors.col(i).norm();
            eigenvectors.col(i) /= norm;
        }
        eigen_pairs.push_back(make_pair(eigenvalues(i),eigenvectors.col(i)));
    }
    sort(eigen_pairs.begin(),eigen_pairs.end(), [](const pair<double,VectorXd> a, const pair<double,VectorXd> b) -> bool {return (a.first > b.first);} );
    for(unsigned int i = 0; i < eigen_pairs.size(); i++){
        eigenvalues(i) = eigen_pairs[i].first;
        c += eigenvalues(i);
        cumulative(i) = c;
        eigenvectors.col(i) = eigen_pairs[i].second;
    }
    transformed.resize(X.rows(),components);

    for(unsigned int i = 0; i < X.rows(); i++){
        for(unsigned int j = 0; j < components; j++){
```

```
            for (int k = 0; k < K.rows(); k++){
                transformed(i,j) += K(i,k) * eigenvectors(k,j);
            }
        }
    }
    cout << "Sorted eigenvalues:" << endl;
    for(unsigned int i = 0; i < eigenvalues.rows(); i++){
        if(eigenvalues(i) > 0){
            cout << "PC " << i+1 << ": Eigenvalue: " << eigenvalues(i);
            printf("\t(%3.3f of variance, cumulative =  %3.3f)\n", eigenvalues(i)/eigenvalues.sum(),cumulative(i)/eigenvalues.sum());
        }
    }
    cout << endl;
    //cout << "Sorted eigenvectors:" << endl << eigenvectors << endl << endl;
    //cout << "Transformed data:" << endl << transformed << endl << endl;
}


void PCA::print(){

    cout << "Input data:" << endl << X << endl << endl;
    cout << "Centered data:"<< endl << Xcentered << endl << endl;
    cout << "Covariance matrix:" << endl << C << endl << endl;
    cout << "Eigenvalues:" << endl << eigenvalues << endl << endl;
    cout << "Eigenvectors:" << endl << eigenvectors << endl << endl;
    cout << "Sorted eigenvalues:" << endl;
    for(unsigned int i = 0; i < eigenvalues.rows(); i++){
        if(eigenvalues(i) > 0){
            cout << "PC " << i+1 << ": Eigenvalue: " << eigenvalues(i);
            printf("\t(%3.3f of variance, cumulative =  %3.3f)\n", eigenvalues(i)/eigenvalues.sum(),cumulative(i)/eigenvalues.sum());
        }
    }
    cout << endl;
    cout << "Sorted eigenvectors:" << endl << eigenvectors << endl << endl;
    cout << "Transformed data:" << endl << X * eigenvectors << endl << endl;
    //cout << "Transformed centred data:" << endl << transformed << endl << endl;

}

void PCA::write_transformed(string file){

    ofstream outfile(file);
    for(unsigned int i = 0; i < transformed.rows(); i++){
        for(unsigned int j = 0; j < transformed.cols(); j++){
            outfile << transformed(i,j);
            if(j != transformed.cols()-1) outfile << ",";
        }
        outfile << endl;
    }
    outfile.close();
    cout << "Written file " << file << endl;

}

void PCA::write_eigenvectors(string file){

    ofstream outfile(file);
    for(unsigned int i = 0; i < eigenvectors.rows(); i++){
```

```
        for(unsigned int j = 0; j < eigenvectors.cols(); j++){
            outfile << eigenvectors(i,j);
            if(j != eigenvectors.cols()-1) outfile << ",";
        }
        outfile << endl;
    }
    outfile.close();
    cout << "Written file " << file << endl;

}
```

Verification - Mahalanobis Distance

The function calcCovarMatrix() calculates the covariance matrix between two vectors. The calculation function cvMahalonobis() is integrated in OpenCV.

```
double CPPPCA::CalMahDistance(Mat matSrc, Mat matTest)
{
    Mat matCovar, matMean;
    CvMat cvmatSrc, cvmatTest, cvmatCovar;
    cvmatSrc = matSrc;
    cvmatTest = matTest;

    calcCovarMatrix(&matSrc, 1, matCovar, matMean, CV_COVAR_NORMAL);
    cvmatCovar = matCovar;

    double dbMahDistance = cvMahalonobis(&cvmatSrc, &cvmatTest, &
        cvmatCovar);

    return dbMahDistance;

}    // end of CPPPCA::CalMahDistance()
```

Verification - Correlation

```
double CPPPCA::GetCorelation(uchar *v1, uchar *v2, int n)
{
    double dSignal = 0, dNoise = 0;
    int i;
    double e1 = 0, e2 = 0, e12 = 0, c1 = 0, c2 = 0;

    //calculate the expectations of V1, V2 and V1 x V2
    for (i = 0; i < n; i++){
        e1  += v1[i];
        e2  += v2[i];
        e12 += v1[i] * v2[i];
    }

    e1  = e1  / n;
    e2  = e2  / n;
    e12 = e12 / n;

    //calulate the variances of R1 and R2
    for (i = 0; i < n; i++){
        c1 += (e1 - v1[i]) * (e1 - v1[i]);
        c2 += (e2 - v2[i]) * (e2 - v2[i]);
    }

    c1 = sqrt(c1 / n);
    c2 = sqrt(c2 / n);
```

```

    //calulate the correlation
    return IsZero(c1) || IsZero(c2)? 0.0 : fabs((e12 - e1 * e2) / (c1 * c2
        ));

}     //end of CPPPCA::GetCorelation()
```
